# Phosphatidylinositol (3,5)-bisphosphate machinery regulates neurite thickness through neuron-specific endosomal protein NSG1/NEEP21

**DOI:** 10.1016/j.jbc.2022.102775

**Published:** 2022-12-07

**Authors:** Lijuan Qi, Chen Sun, Shenqing Sun, Aiqing Li, Qiuming Hu, Yaobo Liu, Yanling Zhang

**Affiliations:** 1 Department of Biochemistry and Molecular Biology, Soochow University Medical College, Suzhou, Jiangsu, China; 2 National Clinical Research Center for Hematologic Diseases, Jiangsu Institute of Hematology, The First Affiliated Hospital of Soochow University, Suzhou, Jiangsu, China; 3 Jiangsu Key Laboratory of Neuropsychiatric Diseases and Institute of Neuroscience, Clinical Research Center of Neurological Disease, The Second Affiliated Hospital of Soochow University, Soochow University, Suzhou, Jiangsu, China

**Keywords:** PtdIns(3,5)P_2_, PIKfyve, NSG1, neurite outgrowth, neurite thickness, APP, amyloid precursor protein, CAD, Cath.a-differentiated cells, div, days in vitro, FBS, fetal bovine serum

## Abstract

Phosphatidylinositol (3,5)-bisphosphate [PtdIns(3,5)P_2_] is a critical signaling phospholipid involved in endolysosome homeostasis. It is synthesized by a protein complex composed of PIKfyve, Vac14, and Fig4. Defects in PtdIns(3,5)P_2_ synthesis underlie a number of human neurological disorders, including Charcot-Marie-Tooth disease, child onset progressive dystonia, and others. However, neuron-specific functions of PtdIns(3,5)P_2_ remain less understood. Here, we show that PtdIns(3,5)P_2_ pathway is required to maintain neurite thickness. Suppression of PIKfyve activities using either pharmacological inhibitors or RNA silencing resulted in decreased neurite thickness. We further find that the regulation of neurite thickness by PtdIns(3,5)P_2_ is mediated by NSG1/NEEP21, a neuron-specific endosomal protein. Knockdown of NSG1 expression also led to thinner neurites. mCherry-tagged NSG1 colocalized and interacted with proteins in the PtdIns(3,5)P_2_ machinery. Perturbation of PtdIns(3,5)P_2_ dynamics by overexpressing Fig4 or a PtdIns(3,5)P_2_-binding domain resulted in mislocalization of NSG1 to nonendosomal locations, and suppressing PtdIns(3,5)P_2_ synthesis resulted in an accumulation of NSG1 in EEA1-positive early endosomes. Importantly, overexpression of NSG1 rescued neurite thinning in PtdIns(3,5)P_2_-deficient CAD neurons and primary cortical neurons. Our study uncovered the role of PtdIns(3,5)P_2_ in the morphogenesis of neurons, which revealed a novel aspect of the pathogenesis of PtdIns(3,5)P_2_-related neuropathies. We also identified NSG1 as an important downstream protein of PtdIns(3,5)P_2_, which may provide a novel therapeutic target in neurological diseases.

PtdIns(3,5)P_2_ is a signaling phospholipid found in the membrane of endosomes/lysosomes. It is synthesized by a ternary protein complex composed of lipid kinase PIKfyve/Fab1, scaffolding protein Vac14, and lipid phosphatase Fig4/Sac3 (1, 2, 3, 4) (Fig. 1*A*). As one of the seven phosphoinositide lipids, PtdIns(3,5)P_2_ plays crucial roles in endomembrane homeostasis, autophagy, ion transport, stress adaptation, and signaling pathways (5, 6, 7). PIKfyve, the kinase for PtdIns(3,5)P_2_ biosynthesis, has become an intensively studied drug target in recent years for treating non-Hodgkin lymphoma (8), multiple myeloma (9), COVID-19 (10), cardiac fibrosis (11), and neurodegenerative diseases (12, 13).

**Figure 1:**
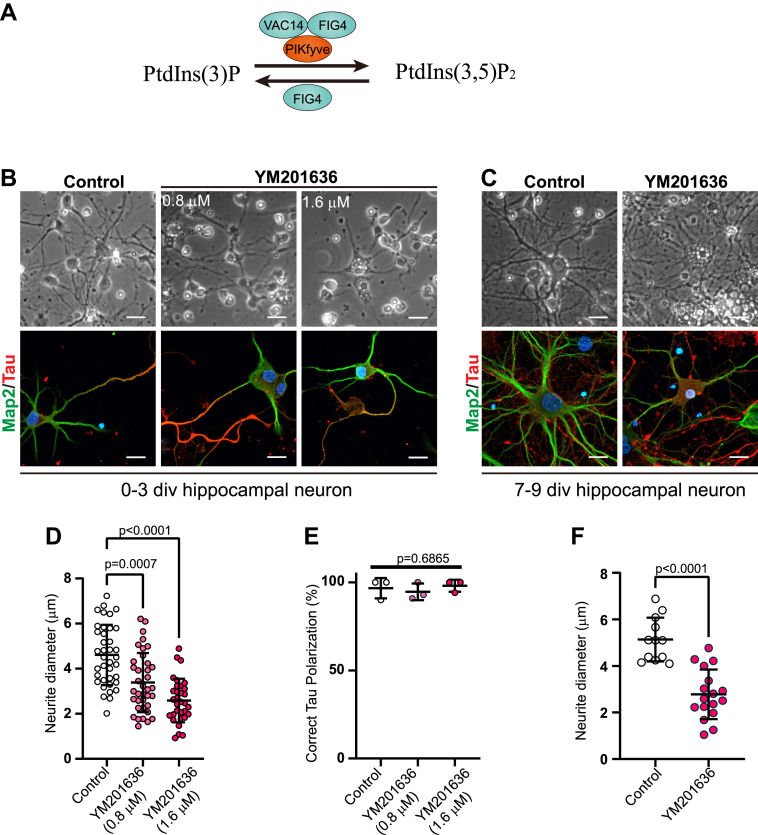
**Inhibition of PtdIns(3,5)P**_**2**_**biosynthesis leads to neurite thinning in primary neurons.***A*, diagram of PtdIns(3,5)P_2_ biosynthesis and turnover pathways. *B* and *C*, cultured mouse hippocampal neurons were treated with DMSO (Control) or PIKfyve inhibitor YM201636 at 0.8 μM or 1.6 μM for 48 h from 1 div to 3 div after plating (*B*) or at 1.6 μM from 7 div to 9 div (*C*). *Top*: Phase contrast microscopy. *Bottom*: Immunofluorescence with dendritic marker anti-Map2 (*green*) and axon marker anti-Tau (*red*). *D*, quantitation of neurite thickness in (*B*). N = 32 to 38 neurons from three independent experiments. Significance was determined by Kruskal-Wallis test followed by Dunn's multiple comparison test adjustment. *E*, percentage of neurons with correct Tau polarization in (*B*). N = 3 independent experiments, 7 to 15 neurons per experiment. Significance was determined by one-way ANOVA. *F*, quantitation of neurite thickness in (*C*). N = 12 to 17 neurons from three independent experiments. Significance was determined by Mann-Whitney test. Scale bars represent 20 μm. Error bars, mean ± SD. div, days in vitro.

The nervous system is particularly susceptible to disruption in the PtdIns(3,5)P_2_ pathway. Deficiencies in the PtdIns(3,5)P_2_ biosynthetic machinery, PIKfyve/Vac14/Fig4, are implicated in a number of human neurological disorders. Mutations in Fig4 were firstly identified to cause the peripheral neuropathy Charcot-Marie-Tooth disease type 4J (14). Later on, other neurological diseases such as Yunis-Varón syndrome (15, 16), familial epilepsy (17), and cerebral hypomyelination (18) were also linked to Fig4 mutations. Similarly, biallelic pathogenic Vac14 variants have been found to underlie child onset progressive dystonia and degeneration in the striatum, substantia nigra, and basal ganglia region (19, 20, 21, 22, 23, 24, 25). It is worth noting that Vac14 variants were also identified in familial parkinsonism disorder (26, 27). Consistently, mouse models deficient in PtdIns(3,5)P_2_ synthesis displayed a range of neurological phenotypes, including spongiform degeneration in the brain, gliosis, hypomyelination, and reduced nerve conduction velocity (3, 14, 28, 29). These findings emphasize the importance of the PtdIns(3,5)P_2_ pathway in the nervous system.

On the cellular level, PtdIns(3,5)P_2_ modulates the trafficking of many essential neuronal proteins. A subpopulation of Vac14 proteins localizes at excitatory synapses in cultured hippocampal neurons (30). Synaptic strength and plasticity can be bidirectionally regulated by levels of PtdIns(3,5)P_2_ (31). Surface levels of AMPA-type glutamate receptors are increased in Vac14^−/−^ and Fig4^−/−^ neurons due to altered internalization and recycling to the plasma membrane. In addition, Vac14 physically interacts with amyloid precursor protein (APP) (32, 33), the central player in Alzheimer's disease, and synphilin-1, an aggregation-prone protein implicated in Parkinson's disease (34). Inhibition of PtdIns(3,5)P_2_ pathway results in the accumulation of APP in early endosomes (33).

NSG1 (also called NEEP21) is a neuron-specific endosomal protein. The expression of NSG1 is mainly restricted to neurons (35, 36). It has been reported that NSG1 is involved in the trafficking of AMPA-type glutamate receptors (GluR1 and GluR2) (35, 37), APP (38), adhesion protein NgCAM/L1 (39), and neurotensin receptors (40). As PtdIns(3,5)P_2_ is also involved in glutamate receptors and APP trafficking, it is conceivable that PtdIns(3,5)P_2_ and NSG1 regulate similar endosomal transport steps.

Neurite thickness is an essential determinant of neuronal function. It has been well established that conduction velocity in axons is proportional to fiber diameter in myelinated axons and the square root of diameter in unmyelinated axons (41, 42). In recent years, it was found that dendrite diameter also plays an important role in excitability (43, 44), action potential amplitude (45), and voltage propagation (46, 47, 48). In the physiological setting, dendrite diameters of central auditory neurons are correlated with their frequency response (49). In the pathological setting, exposure of hippocampal neurons to amyloid Aβ results in a decrease in dendrite diameters (50), which could contribute to defective information processing in Alzheimer's disease. However, the regulation of neurite thickness remains little understood.

In this study, we found that PtdIns(3,5)P_2_ regulates neurite thickness through NSG1/NEEP21. Suppressing either PtdIns(3,5)P_2_ pathway or NSG1 expression resulted in thinner neurites. Proteins in the PtdIns(3,5)P_2_ machinery colocalized and physically interacted with NSG1. Perturbation of PtdIns(3,5)P_2_ dynamics led to the mislocalization of NSG1. Importantly, overexpression of NSG1 rescued the neurite thickness defect in neurons defective of PtdIns(3,5)P_2_ synthesis. This study revealed a novel role of PtdIns(3,5)P_2_ in neuron morphology and provided a novel target pathway for treating PtdIns(3,5)P_2_-related neuropathies.

## Results

### Defective PtdIns(3,5)P_2_ biosynthesis results in decreased neurite diameters

In previous studies, we noticed that neurites of PtdIns(3,5)P_2_-impaired neurons such as Vac14^−/−^ neurons were often thinner than WT controls (28), suggesting that PtdIns(3,5)P_2_ is possibly involved in regulating neurite thickness. To examine this possibility, we investigated the role of PtdIns(3,5)P_2_ in neuron development by inhibiting PIKfyve. PIKfyve is the only lipid kinase that synthesizes PtdIns(3,5)P_2_, so PIKfyve inhibitors are known to abolish PtdIns(3,5)P_2_ almost completely (29). Mouse hippocampal neurons were cultured *in vitro* and treated with a selective PIKfyve inhibitor YM201636 (51). Consistent with previous observations (30), swollen vacuoles were observed in both cell bodies (soma) and neurites in YM201636-treated neurons (Fig. 1, *B* and *C*). Such vacuolation is a hallmark phenotype of PtdIns(3,5)P_2_-deficient cells (52). When neurons were exposed to YM201636 during early development, from 0 days *in vitro* (div) to 3 div after plating, neurite outgrowth was impaired, forming shorter and thinner neuronal processes (Fig. 1, *B* and *D*). Meanwhile, axon specification, an event during early development characterized by the selection of only one of the neurites to become the axon (53), was not affected by YM201636. The axon marker, Tau, was still restricted to only one neurite in YM201636-treated neurons at 3 div, indicating that PtdIns(3,5)P_2_ was not involved in axon/dendrite polarization (Fig. 1, *B* and *E*). The effect of PtdIns(3,5)P_2_ abolishment was also examined during late neuron development (7 div to 9 div), when neurites were already formed. In YM201636-treated older cultures, dendrites labeled by Map2 were noticeably thinner, and axon networks marked by Tau were much sparser (Fig. 1, *C* and *F*). Taken together, these results suggest that PtdIns(3,5)P_2_ is involved in regulating and maintaining neurite morphology, particularly neurite thickness, but not axon/dendrite polarization.

**Figure 2:**
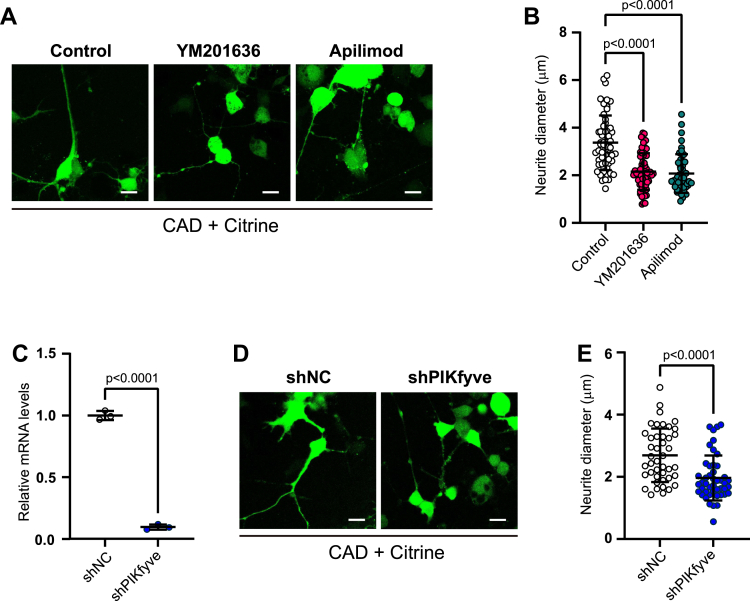
**Inhibition of PtdIns(3,5)P**_**2**_**biosynthesis results in neurite thinning in CAD cells.***A*, CAD cells were transfected with volume marker Citrine, differentiated, and treated with DMSO (Control), 1.6 μM YM201636, or 1 μM apilimod for 12 h. *B*, quantitation of neurite diameters in (*A*). N = 45 to 65 cells from three independent experiments. Significance was determined by Kruskal-Wallis test followed by Dunn's multiple comparison test. *C*, CAD cells were infected with lentiviruses expressing nontargeting control shRNA (shNC) or shRNA against mouse PIKfyve gene (shPIKfyve) and selected with 2 μg/ml puromycin. The expression of PIKfyve mRNA was determined by qRT-PCR. N = 3 independent experiments. Significance was determined by Student’s *t* test. *D*, stable shPIKfyve and shNC CAD cells were transfected with Citrine and differentiated for 24 h. *E*, quantitation of neurite diameters in (*D*). N = 43 to 46 cells from three independent experiments. Significance was determined by Mann-Whitney test. Scale bars represent 20 μm. Error bars, mean ± SD. CAD, Cath.a-differentiated cells.

It was reported that inhibition of PIKfyve could result in cell death in primary neuron cultures (54). To exclude the effect of neuronal viability on neurite morphology, we made use of Cath.a-differentiated cells (CAD) cells, a neuron cell line derived from catecholaminergic neurons in the central nervous system (55). Neurite growth in CAD cells can be conveniently initiated by serum withdrawal from culturing media, which provides a unique system of “on-demand” neurite growth. A fluorescent protein, Citrine, was transfected into CAD cells to serve as a volume marker. When treated by YM201636, or another structurally distinct PIKfyve inhibitor apilimod (56), CAD neurites were significantly thinner than the controls (Fig. 2, *A* and *B*), consistent with the results in hippocampal neurons (Fig. 1, *D* and *F*). Moreover, to exclude possible side effects of chemical inhibitors, PIKfyve expression was specifically stably knocked down with lentivirus-mediated shRNA expression (shPIKfyve) in CAD cells (Fig. 2*C*). After differentiation, stable shPIKfyve cells actively developed much thinner neurites than control cells with empty vector infection (shNC) (Fig. 2, *D* and *E*). Note that neurite growth occurred after PIKfyve knockdown, excluding cell viability as the explanation for the thinner neurites. Collectively, these results demonstrate that PIKfyve inhibition resulted in thinner neurites in both primary hippocampal neurons and CAD neurons, suggesting that regulation of neurite thickness by PtdIns(3,5)P_2_ is a common mechanism in multiple neuronal types.

### NSG1 colocalizes and physically interacts with PtdIns(3,5)P_2_ machinery

**Figure 3:**
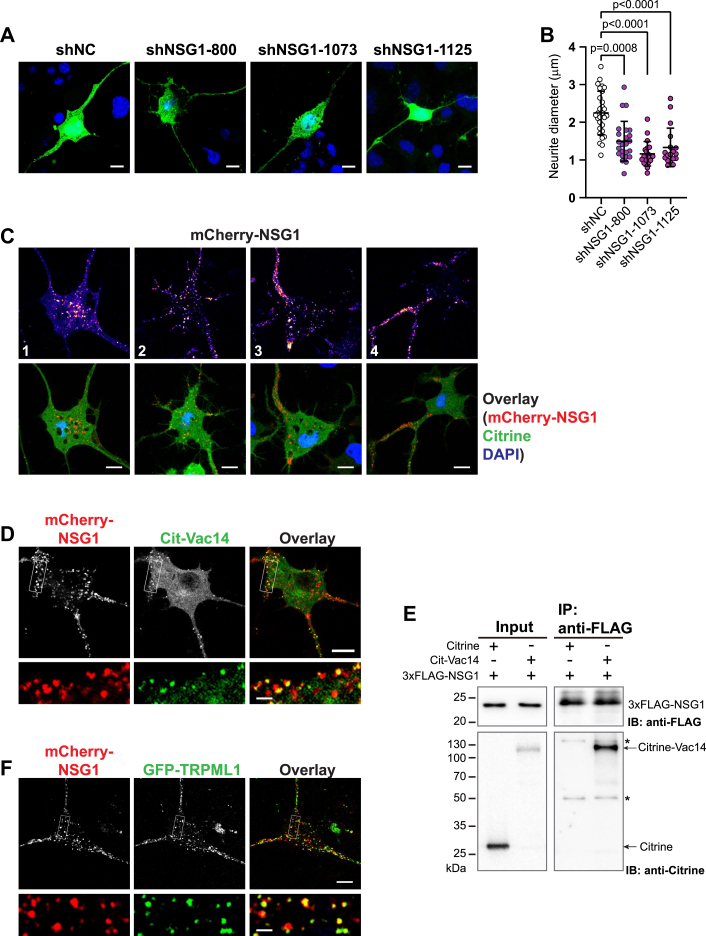
**NSG1 colocalizes with PtdIns(3,5)P**_**2**_**machinery.***A*, knockdown of NSG1 expression led to neurite thinning in CAD cells. CAD cells were transiently transfected with plasmids expressing GFP and three independent shRNAs against mouse NSG1 (shNSG1) or nontargeting shRNA (shNC), followed by differentiation for 24 h. Scale bars represent 10 μm. *B*, quantitation of neurite thickness in (*A*) for shNC and shNSG1-expressing cells. N = 18 to 29 cells from three independent experiments. Significance was determined by Kruskal-Wallis test followed by Dunn's multiple comparison test. *C*, distribution of mCherry-NSG1 among the soma and neurites. CAD cells were cotransfected with Citrine and mCherry-NSG1. *Top*: mCherry-NSG1 signals displayed with “Fire” LUT. *Bottom*: Overlay of mCherry-NSG1 and the volume marker Citrine. Scale bars represent 10 μm. *D*, CAD cells were cotransfected with mCherry-NSG1 and Citrine-Vac14. *Yellow* puncta in the overlay channel indicate colocalization. Scale bars represent 10 μm for the *upper panel* and 2 μm for the zoomed insets. *E*, Cit-Vac14 and 3xFLAG-NSG1 were in the same physical complex. HEK293T cells were cotransfected with 3xFLAG-NSG1 and Cit-Vac14 or Citrine control. Cell extracts were pulled down by anti-FLAG resin and immune-blotted with anti-FLAG and anti-Citrine. *Stars* (∗) denote nonspecific bands in the immunoprecipitates. *F*, CAD cells were cotransfected with mCherry-NSG1 and GFP-TRPML1. Scale bars represent 10 μm for the *upper panel* and 2 μm for the zoomed insets. All images in panels *C*, *D*, and *E* were single z-plane images taken with a Leica confocal microscope. Error bars, mean ± SD. CAD, Cath.a-differentiated cells.

NSG1/NEEP21 is a neuron-specific membrane protein involved in the trafficking of a number of physiologically critical cargoes. We noticed that there is a significant overlap in phenotypes regulated by PtdIns(3,5)P_2_ or NSG1, including AMPA receptor–mediated synaptic plasticity (37) and APP processing (38). Thus, we set out to determine the relationship between NSG1 and PtdIns(3,5)P_2_ in regulating neurite thickness. Firstly, NSG1 expression was knocked down in CAD cells by three independent shRNA constructs. Neurite thinning was observed in all of them (Fig. 3, *A* and *B*), similar to shPIKfyve cells. This phenotype could be rescued when an mCherry-tagged NSG1 (mCherry-NSG1) was reintroduced to the NSG1 knockdown cells (Fig. S1, *A–C*). This result suggests that both PtdIns(3,5)P_2_ and NSG1 regulate neurite thickness.

**Figure 4:**
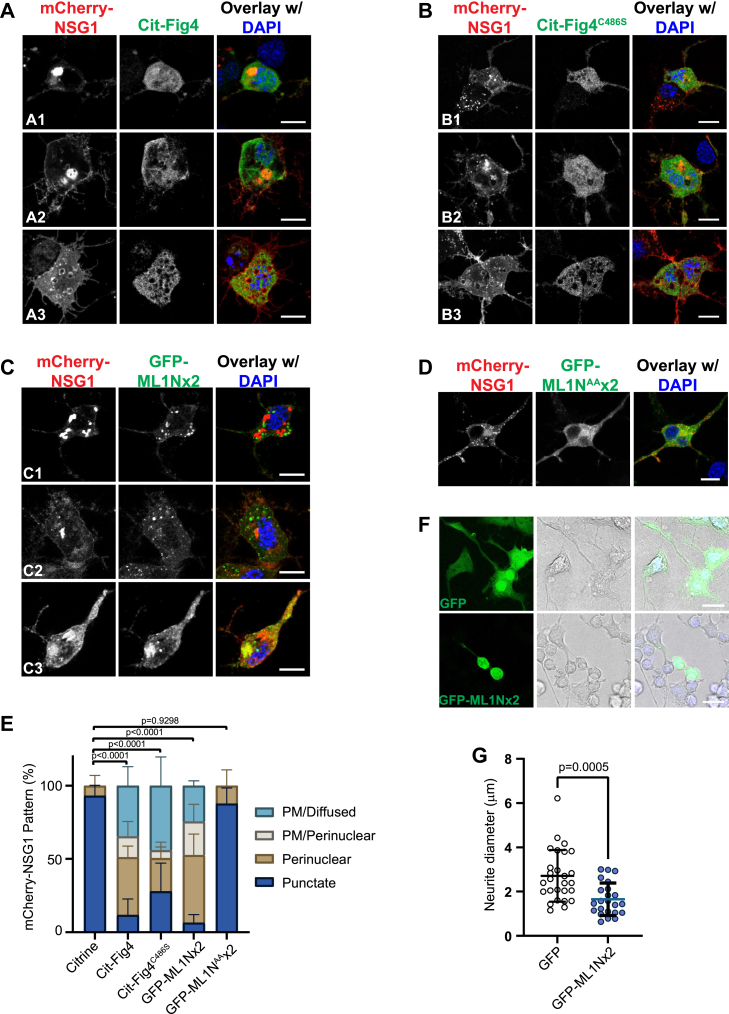
**NSG1 proteins are mislocalized when PtdIns(3,5)P**_**2**_**dynamics is perturbed.***A* and *B*, CAD cells were cotransfected with mCherry-NSG1 and Citrine-Fig4 (Cit-Fig4) (*A*) or Cit-Fig4^C486S^ mutant (*B*). Multiple mislocalization patterns of mCherry-NSG1 were observed: concentrated in the perinuclear region (A1, B1), both perinuclear region and the plasma membrane surface (A2, B2), or diffused (A3, B3). *C* and *D*, CAD cells were cotransfected with mCherry-NSG1 and GFP-ML1N×2 (*C*) or GFP-ML1N^AA^×2 mutant (*D*). Mislocalization patterns of mCherry-NSG1 in the perinuclear region (C1), both perinuclear region and the plasma membrane surface (C2), or the plasma membrane/diffused (C3) were observed in cells GFP-ML1N×2 expression, but not GFP-ML1N^AA^×2 expression. *E*, quantitation of the percentage of mCherry-NSG1 mislocalization patterns in (*A–D*) compared to control cells expressing Citrine control. N = 3 to 4 independent experiments, 16 to 39 cells per experiment. Citrine is a variant of GFP. Significance was determined using the percentage of punctate mCherry-NSG1 cells by one-way ANOVA followed by Dunnett's multiple comparison test. *F*, overexpression of GFP-ML1N×2 leads to neurite thinning. CAD cells were cotransfected with EGFP or GFP-ML1N×2. *G*, quantitation of neurite thickness in (*F*). N = 23 to 27 cells from three independent experiments. Significance was determined by Mann-Whitney test. Scale bars represent 10 μm in (*A–D*) and 25 μm in (*F*). Some neurons spanned multiple z-planes and required projections of max intensity, including images used for A1, A3, B1, and *D*, while other images were single z-planes. Error bars, mean ± SD. CAD, Cath.a-differentiated cells; PM, plasma membrane surface.

The localization of NSG1 was determined with mCherry-NSG1 in CAD cells. Consistent with previous reports (35), mCherry-NSG1 localized to punctate endosomal structures (Video S1 and Fig. 3*C*). Notably, mCherry-NSG1 puncta exhibited active long-range and bidirectional movements along the neurites, as well as between neurites and soma (Video S1). At the steady state, the distribution of mCherry-NSG1 between soma and neurites varied among individual cells, ranging from mainly soma-localized (Fig. 3, *C*1) to almost exclusively neurite-localized (Figure 3, Figure 4), with many intermediate states in between. Similar patterns were also observed in PC12 cells, another commonly used neuronal cell line (Fig. S1*D*). These results suggest an active transport route for NSG1-positive vesicles between soma and neurites.

To determine the spatial relationship between NSG1 and PtdIns(3,5)P_2_ machinery, mCherry-NSG1 was coexpressed with Citrine-tagged Vac14 (Cit-Vac14). Vac14 is an essential component in the PtdIns(3,5)P_2_ machinery that forms a star-shaped pentameric scaffold and nucleates PIKfyve and Fig4 in the same assembly (4). A significant fraction of punctate Vac14 (57.8 ± 0.07%, n = 9) was found colocalizing with mCherry-NSG1 (Fig. 3*D*), indicating that both proteins were on the same endosomes. Next, we determined if they physically interact. HEK293T cells were cotransfected with 3×FLAG-tagged NSG1 and Cit-Vac14 or Citrine control. NSG1 was pulled down with anti-FLAG resin. Cit-Vac14, but not Citrine, was found in the precipitates (Fig. 3*E*), suggesting that NSG1 and Vac14 were in the same physical complex.

In addition, we determined the colocalization between NSG1 and TRPML1, an endolysosome-localized cation channel activated by PtdIns(3,5)P_2_ (57). GFP-TRPML1 puncta displayed an extensive overlap (59.2 ± 0.05%, n = 14) with mCherry-NSG1 (Fig. 3*F* and Video S2) in CAD cells. This colocalization was especially prominent when mCherry-NSG1 was mainly neurite-localized (Video S2). The colocalization between mCherry-NSG1 and GFP-TRPML1 was also evident when they were coexpressed in nonneuronal HeLa cells (Fig. S1*E*). Together, these results suggest that NSG1 colocalizes and physically interacts with proteins involved in the biosynthesis and signaling of PtdIns(3,5)P_2_, confirming the important relationship between NSG1 and PtdIns(3,5)P_2_ pathway.

It has been reported that NSG1 is a type II transmembrane protein that travels rapidly from Golgi to the plasma membrane, followed by internalization into the endocytosis pathway (58). We assessed whether mCherry-NSG1 functions with PtdIns(3,5)P_2_ at specific endocytic stages. Three endocytic markers, EEA1 (early endosomes), CD63 (late endosomes), and LAMP1 (late endosomes/lysosomes) were used to mark various endosomes (Fig. S2). Consistent with the previous report (58), mCherry-NSG1 could be found throughout the endocytic pathway, with the highest colocalization with late endosomes (Fig. S2, *B* and *D*). Furthermore, mCherry-NSG1 endosomes that colocalized with Cit-Vac14 or TRPML1 had a similar distribution of endocytic markers as those of overall mCherry-NSG1 puncta (Fig. S2, *B* and *D*), suggesting that the colocalization was not restricted to a particular endocytic stage.

### Perturbation of PtdIns(3,5)P_2_ dynamics mislocalizes NSG1

We initially hypothesized that Fig4, the phosphatase within the PtdIns(3,5)P_2_ biosynthesis complex, also colocalized with NSG1. Unexpectedly, the punctate localization of mCherry-NSG1 proteins was largely lost in Cit-Fig4–expressing cells (Fig. 4, *A* and *E*). When Cit-Fig4 was overexpressed, mCherry-NSG1 proteins were either concentrated in the perinuclear region, on the cell surface, or appeared diffused (Fig. 4, *A* and *E*), suggesting that mCherry-NSG1 proteins were trapped in the endoplasmic reticulum/Golgi step or at the plasma membrane before internalization into endosomes. In Cit-Fig4–overexpressed cells, neurites were often ill-formed and developed curly spiny branches instead of a stable shaft (Fig. 4, *A*2 and *A*3). CAD cells in the same microscopic field with no Cit-Fig4 coexpression displayed normal endosomal localization of mCherry-NSG1 and proper neurite morphology (Compare arrowheads with arrows in Fig. S3*A*). We also repeated this experiment in PC12 cells and observed similar mislocalization patterns of mCherry-NSG1 upon Cit-Fig4 overexpression (Fig. S3*B*).

Fig4 is a unique member in the PIKfyve/Vac14/Fig4 complex since it harbors phosphatase activity and could dephosphorylate PtdIns(3,5)P_2_ (59, 60). In addition, Fig4 also acts as a protein phosphatase and stimulates the kinase activity of PIKfyve (Fig. 1*A*) (4). Therefore, Fig4 functions in both biosynthesis and turnover of PtdIns(3,5)P_2_ (4, 61, 62). It was possible that overexpression of Fig4 led to reduced PtdIns(3,5)P_2_ levels and hence NSG1 mislocalization. To explore this possibility, we employed a phosphatase-dead Fig4^C486S^ mutant (62), where the catalytic cysteine was mutated to serine. Nonetheless, when the Cit-Fig4^C486S^ mutant was coexpressed with mCherry-NSG1, mislocalization of mCherry-NSG1 was still observed in both CAD cells (Fig. 4, *B* and *E*) and PC12 cells (Fig. S3*C*), albeit with somewhat lower efficiency, suggesting that the phosphatase activity of Fig4 was not required for NSG1 mislocalization. Rather, overexpression of Fig4 probably perturbed the assembly stoichiometry of PtdIns(3,5)P_2_ machinery and impaired the dynamics of PtdIns(3,5)P_2_, which in turn caused NSG1 mislocalization.

GFP-ML1N×2 is a tandem repeat of the N-terminal segment of TRPML1 that binds to PtdIns(3,5)P_2_ and has been used as a probe for PtdIns(3,5)P_2_ localization (63). Like Cit-Fig4, we found that overexpression of GFP-ML1N×2 also caused the mislocalization of mCherry-NSG1 (Fig. 4, *C* and *E*). CAD cells in the same field with no ML1N×2 coexpression had normal punctate mCherry-NSG1 (Compare arrowheads with arrows in Fig. S4*A*). We reasoned that ML1N×2 probably masked the accessibility of PtdIns(3,5)P_2_ and thus acted as a dominant-negative protein here. To test this hypothesis, mCherry-NSG1 was coexpressed with GFP-ML1N^AA^×2 mutant, which had residues R^61^ K^62^ mutated to alanines and lost binding to PtdIns(3,5)P_2_ (63). In these cells, mCherry-NSG1 had normal punctate endosomal localization (Fig. 4, *D* and *E*), suggesting that the PtdIns(3,5)P_2_-binding ability of ML1N×2 was necessary for the mislocalization of mCherry-NSG1. Neurites in GFP-ML1N×2–expressing cells were often thinner (Fig. 4, *C1*). Indeed, expressing GFP-ML1N×2 alone, without coexpressing mCherry-NSG1, was sufficient to induce the neurite thinning phenotype (Fig. 4, *F* and *G*), which supported our previous observation that proper PtdIns(3,5)P_2_ signaling is essential for neurite thickness.

Additionally, we examined the effect of reducing the duration of exogenously expressing Fig4 and ML1N×2. In a different transfection scheme, mCherry-NSG1 was transfected first, followed by neuron differentiation and a brief expression of Citrine-Fig4 or GFP-ML1N×2 (Fig. S5*A*). In this setting, mCherry-NSG1 proteins were punctate (Fig. S5, *B–C*), although neurites started to show short curly branches in Cit-Fig4–expressing cells (Fig. S5*B*). Importantly, mCherry-NSG1 colocalized with briefly-expressed GFP-ML1N×2 (Fig. S5*C*), both in the soma and in neurites, in agreement with previous results on the colocalization between mCherry-NSG1 and PtdIns(3,5)P_2_ machinery.

### Inhibition of PtdIns(3,5)P_2_ biosynthesis traps NSG1 in EEA1-positive endosomes

**Figure 5:**
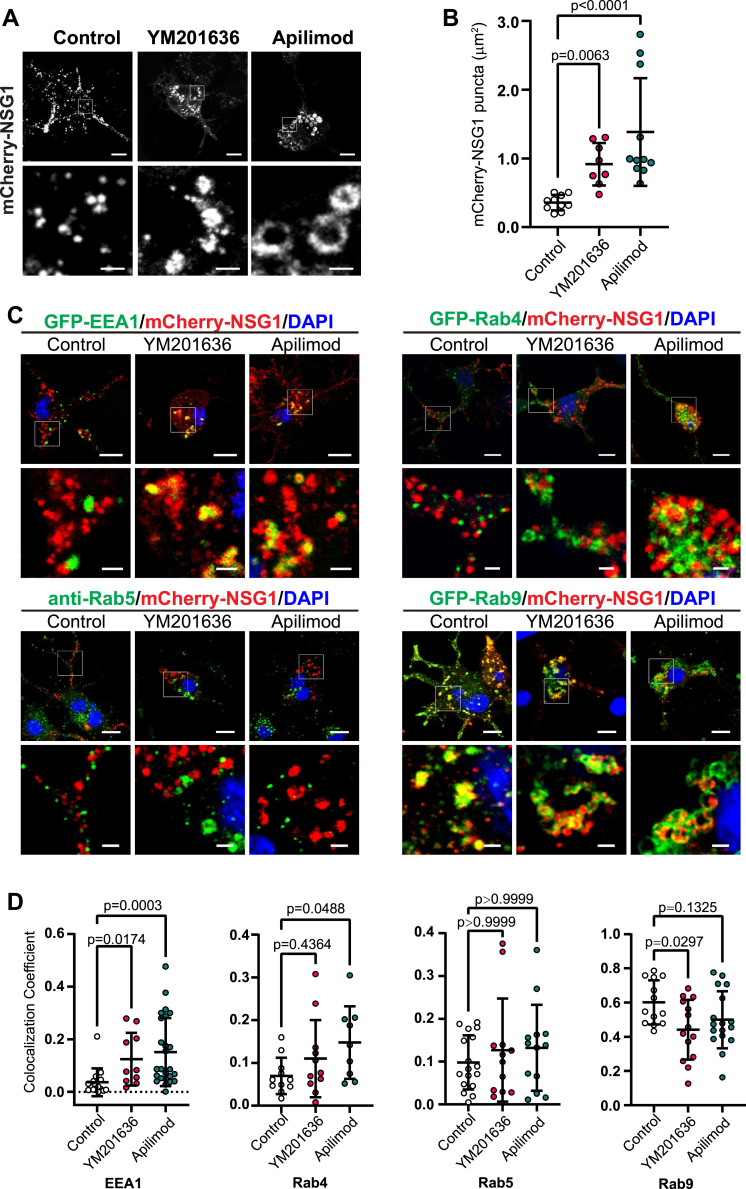
**NSG1 protein transport is delayed in EEA1 endosomes when PtdIns(3,5)P**_**2**_**biosynthesis is inhibited.***A*, mCherry-NSG1 vesicles were enlarged in PIKfyve inhibitor–treated cells. CAD cells with mCherry-NSG1 expression were treated with DMSO (Control), 1.6 μM YM201636, or 1 μM apilimod for 12 h. Scale bars represent 10 μm for the *upper panel* and 2 μm for the zoomed insets. *B*, quantitation of the size of mCherry-NSG1 vesicles in (*A*). N = 8 to 11 cells, with ∼40 to 200 vesicles per cell. The average vesicle size of each cell was calculated. Significance was determined by Kruskal-Wallis test followed by Dunn's multiple comparison test. *C*, CAD cells transfected with mCherry-NSG1 were treated as in (*A*). Endocytic vesicles were labeled by cotransfection with GFP-EEA1, GFP-Rab4, GFP-Rab9, or immunostained with anti-Rab5. Scale bars represent 10 μm for the main panels and 2 μm for the zoomed insets. *D*, quantitation of colocalization between mCherry-NSG1 and endocytic markers in (*C*). N = 10 to 25 cells for EEA1, 9 to 11 cells for Rab4, 12 to 17 cells for Rab5, and 13 to 17 cells for Rab9, from three independent experiments. Significance was determined by Kruskal-Wallis test followed by Dunn's multiple comparison test. Error bars, mean ± SD. All images were single z-plane. CAD, Cath.a-differentiated cells.

The results above demonstrated that mCherry-NSG1 is sensitive to perturbations in the PtdIns(3,5)P_2_ pathway. To further investigate the effect of PtdIns(3,5)P_2_ levels on NSG1 localization, we directly inhibited PtdIns(3,5)P_2_ synthesis with either YM201636 or apilimod in CAD cells transfected with mCherry-NSG1. NSG1-positive endosomes were found enlarged (Fig. 5, *A* and *B*), suggesting defective endosome fission or increased endosome fusion. The enlargement occurred around 30 min to 1 hour after the application of PIKfyve inhibitors (Videos S3 and S4). Similarly, stable shPIKfyve CAD cells transfected with Cherry-NSG1 also showed enlargement of NSG1-positive endosomes (Fig. S6, *A–B*), although the defects in shPIKfyve neurons seemed milder: mCherry-NSG1 endosomes were mostly absent from YM201636 or apilimod-treated neurites (Fig. 5*A*) but still present in shPIKfyve neurites (Fig. S6*A*). This was probably due to residual PIKfyve activity in shPIKfyve cells. These results suggest that PtdIns(3,5)P_2_ is necessary for NSG1 trafficking in endosomes.

To further elucidate the trafficking defects of NSG1 in PtdIns(3,5)P_2_-deficient neurons, we colabeled mCherry-NSG1 with an array of endocytic markers: EEA1 (early endosomes), Rab4 (recycling endosomes), Rab5 (early endosomes), Rab9 (transport between endosomes and trans-golgi network) (Fig. 5, *C* and *D*). Consistent with previous reports (58), mCherry-NSG1 had limited colocalization with EEA1, Rab4, or Rab5 under normal conditions (Fig. 5, *C* and *D*). Interestingly, the colocalization between NSG1 and Rab9 was very significant (Fig. 5*D*), which was consistent with reports that Rab9 and its effector p40 physically interact with the PIKfyve/Vac14 complex (64, 65). After PIKfyve inhibitor treatment, mCherry-NSG1 endosomes had significantly increased colocalization with EEA1 (Manders’ Coefficients from 0.03 to ∼0.12, also see Videos S5–S7), while the colocalization with Rab5 did not change much (Fig. 5, *C* and *D*). A slight increase was observed in the colocalization of NSG1 and Rab4 after PIKfyve inhibitor exposure; however, it was only statistically significant for apilimod but not YM201636 (Fig. 5*D*). The overlap between NSG1 and Rab9 trended downwards after inhibitor treatment, although this decrease was only significant in YM201636 but not apilimod (Fig. 5*D*). Collectively, these results suggested that NSG1 transport is delayed in EEA1-positive endosomes when PtdIns(3,5)P_2_ biosynthesis is impaired.

It was intriguing that direct inhibition of PtdIns(3,5)P_2_ biosynthesis led to less severe NSG1 mislocalization than overexpression of Fig4 or ML1N×2 (Fig. 4), which implies that the dynamics of PtdIns(3,5)P_2_ could be more important than absolute levels of PtdIns(3,5)P_2_ *per se*, at least for NSG1 trafficking. We did notice that, in selected cells with shPIKfyve knockdown (Fig. S6*C*) or PIKfyve inhibitor treatment (Fig. S7*A*), mCherry-NSG1 was mislocalized to the perinuclear region or the surface, similar to cells overexpressing of Fig4 or ML1N×2 (Fig. 4). The perinuclear localization of mCherry-NSG1 proteins overlapped with a Golgi marker, GFP-Rab6, consistent with a blockage at the Golgi step (Fig. S7*B*).

### NSG1 rescues neurite thickness phenotype in PtdIns(3,5) P_2_-impaired neurons

**Figure 6:**
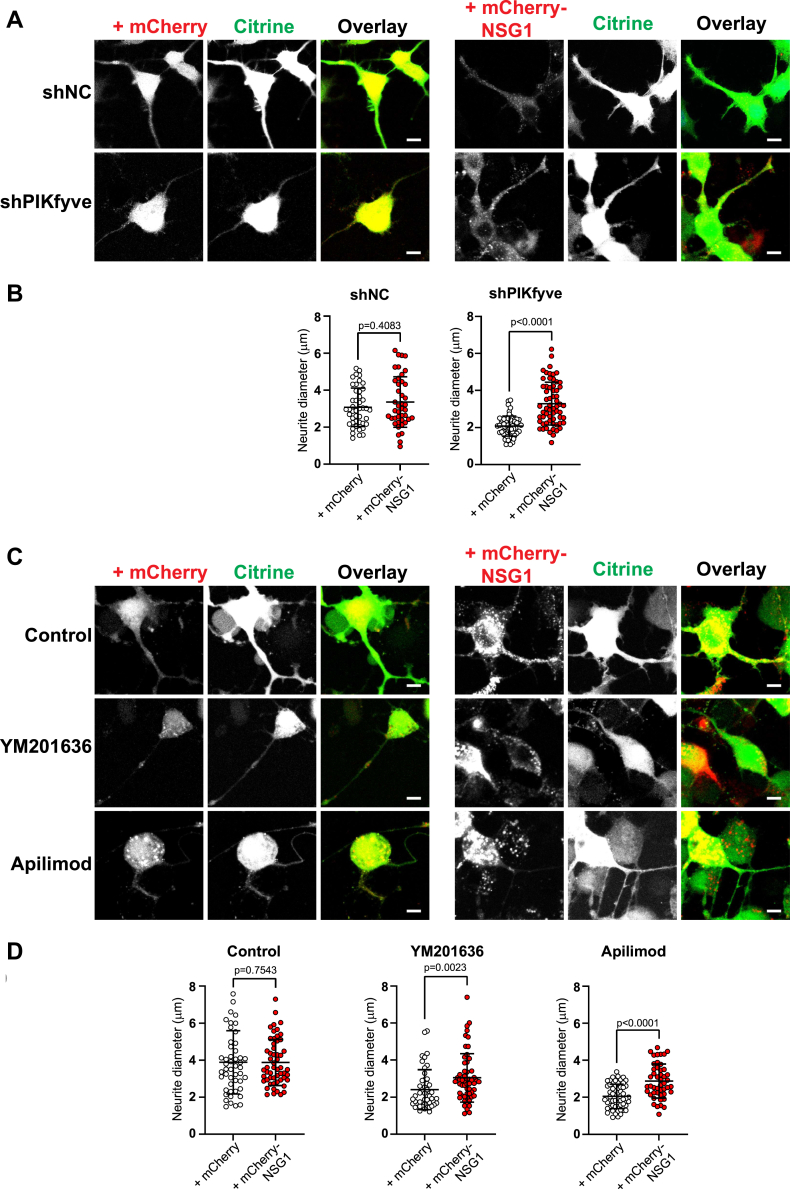
**Overexpression of mCherry-NSG1 rescues defective neurite thickness in PtdIns(3,5)P**_**2**_**-impaired CAD cells.***A*, shNC and shPIKfyve CAD cells were transfected with Citrine (volume marker) and mCherry-NSG1 or mCherry. *B*, quantitation of neurite thickness in (*A*). N = 42 to 62 cells from three independent experiments. Significance was determined by Mann-Whitney test. *C*, CAD cells were transfected with Citrine and mCherry-NSG1 or mCherry, then treated with DMSO (Control), 1.6 μM YM201636, or 1 μM apilimod for 12 h. *D*, Quantitation of neurite thickness in (*C*). N = 46 to 56 cells from three independent experiments. Significance was determined by Mann-Whitney test. Scale bars represent 10 μm. Error bars, mean ± SD. CAD, Cath.a-differentiated cells.

In experiments above, we noted that CAD cells overexpressing mCherry-NSG1 generally had thicker neurites than normal CAD cells when exposed to PIKfyve inhibitors (Fig. 2), suggesting that, even though some of the mCherry-NSG1 proteins were trapped in early endosomes in PtdIns(3,5)P_2_-impaired neurons, additional expression of NSG1 molecules could functionally overcome this traffic delay. To test this notion, we transfected shNC and shPIKfyve CAD cells with mCherry or mCherry-NSG1, together with Citrine as a volume marker. In shPIKfyve cells transfected with mCherry control, neurite diameters were ∼2.1 μm on average (Fig. 6, *A* and *B*). However, with mCherry-NSG1 expression, the average neurite diameter was increased to ∼3.3 μm (Fig. 6, *A* and *B*). In addition, we transfected normal CAD cells with Citrine and mCherry or mCherry-NSG1, followed by treatment with PIKfyve inhibitors. Similarly, mCherry-NSG1 overexpression increased neurite diameters to 126% and 140% in YM201636- or apilimod-treated cells, respectively (Fig. 6, *C* and *D*). These results demonstrated that increased NSG1 expression could alleviate neurite thickness defects in both PIKfyve shRNA knockdown and PIKfyve inhibitor–treated CAD cells.

**Figure 7:**
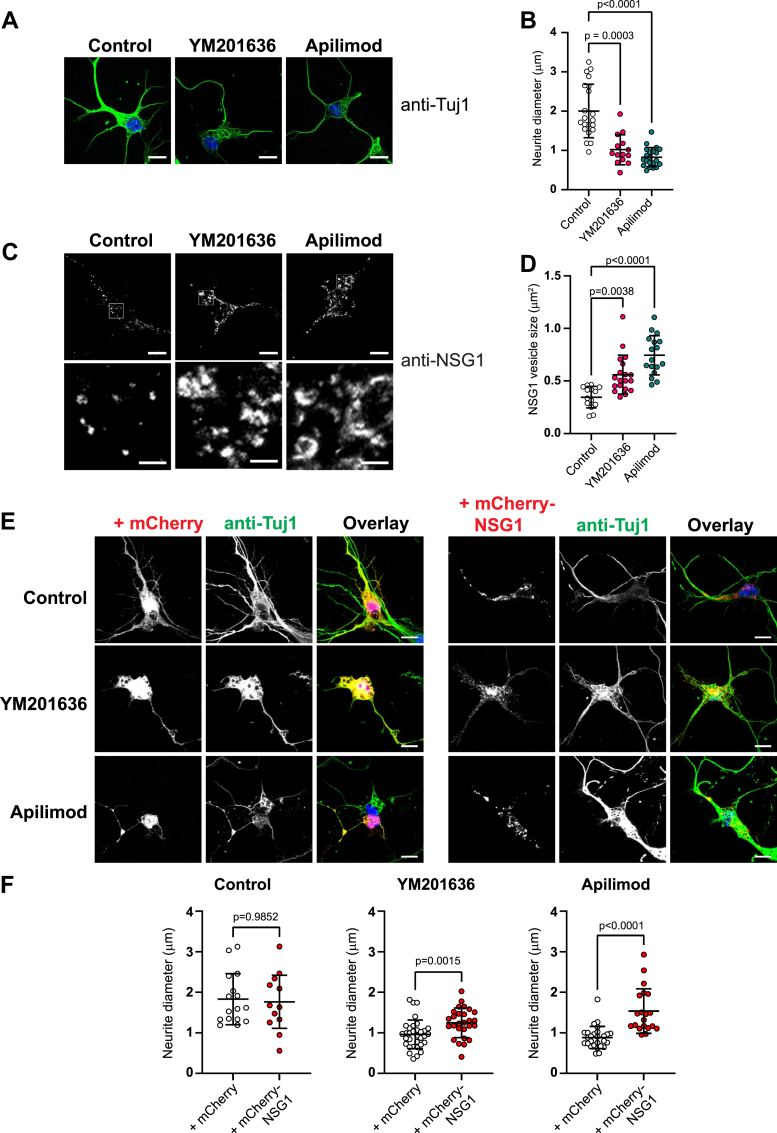
**Overexpression of mCherry-NSG1 rescues neurite thinning in PtdIns(3,5)P**_**2**_**-impaired primary cortical neurons.***A*, primary cortical neurons were treated with DMSO (Control), 1.6 μM YM201636, or 1 μM apilimod for 12 h before fixation and labeled with antibody to Tuj1. Scale bars represent 10 μm. *B*, quantitation of neurite thickness in (*A*). N = 14 to 21 cells from three independent experiments. Significance was determined by Kruskal-Wallis test followed by Dunn's multiple comparison test. *C*, primary cortical neurons were treated with DMSO (Control), 1.6 μM YM201636, or 1 μM apilimod for 12 h before fixation and labeled with antibody to NSG1. Scale bars represent 10 μm for the *upper panel* and 2 μm for the zoomed insets. *D*, quantitation of NSG1 puncta sizes in (*C*). N = 14 to 19 cells from three independent experiments. Significance was determined by Kruskal-Wallis test followed by Dunn's multiple comparison test. *E*, primary cortical neurons were transfected with mCherry-NSG1 or mCherry for 24 h, then treated with DMSO (Control), 1.6 μM YM201636, or 1 μM apilimod for 12 h before fixation and labeled with antibody to Tuj1. Scale bars represent 10 μm. *F*, quantitation of neurite thickness in (*E*). N = 16 to 33 cells from three independent experiments. Significance was determined by Mann-Whitney test. Error bars, mean ± SD.

Next, the functional link between NSG1 and PtdIns(3,5)P_2_ was examined in primary neurons. We utilized mouse neurons from the layer V neocortex, one of the brain regions with the highest NSG1 expression during development (66). Neurites were delineated by antibody to Tuj1 (neuronal-specific class III beta-tubulin). Similar to hippocampal neurons and CAD cells, treatment with PIKfyve inhibitors significantly reduced neurite thickness in cortical neurons from ∼2.1 μm down to ∼1.0 μm (Fig. 7, *A* and *B*). In addition, endogenous NSG1 vesicles were enlarged by both PIKfyve inhibitors (Fig. 7, *C* and *D*). Most importantly, overexpression of mCherry-NSG1 increased the neurite diameter by 30% and 74% in YM201636- or apilimod-treated neurons, respectively (Fig. 7, *E* and *F*), in agreement with results in CAD cells. Collectively, these results suggest that NSG1 is a downstream target of PtdIns(3,5)P_2_ signaling for maintaining the homeostasis of neurite thickness.

## Discussion

Neurons are highly susceptible to disruption in the endosome/lysosome system (67, 68). Genetic variants of endosomal proteins such as BIN1 (69), SORL1 (70), CD2AP (71) have been implicated in Alzheimer’s disease. Similarly, mutations in Vps35 (72), LRRK2 (73), and RME8 (74) are associated with Parkinson’s disease. In recent years, an increasing number of reports have shown that dysregulation of signaling lipid PtdIns(3,5)P_2_ underlie a range of human neurological disorders, such as Charcot-Marie-Tooth disease, Yunis-Varón syndrome, familial epilepsy, progressive dystonia, and parkinsonism. In addition, one computational analysis associated a Vac14 variant with Alzheimer’s disease and bipolar disorder (75). These findings point to the importance of PtdIns(3,5)P_2_ in multiple human neuropathies. Hence, the study of PtdIns(3,5)P_2_ in neurons is of great importance to understand the etiology of various neuropathies and identify new therapeutic targets.

In this study, we described the relationship between PtdIns(3,5)P_2_ machinery and a neuron-specific endosomal protein NSG1. We found that neurite thickness is regulated and maintained by PtdIns(3,5)P_2_ and NSG1. NSG1 colocalized and physically interacted with Vac14, the scaffolding protein for PtdIns(3,5)P_2_ biosynthesis. The transport of NSG1 within neurons required proper PtdIns(3,5)P_2_ dynamics. Importantly, overexpression of NSG1 rescued neurite thinning in PtdIns(3,5)P_2_-deficient neurons, suggesting that NSG1 is downstream of PtdIns(3,5)P_2_ function and that activation of NSG1 function could serve as a new target for PtdIns(3,5)P_2_-related neuropathy. It remains to be investigated whether NSG1 could also rescue other PtdIns(3,5)P_2_-related neuronal defects such as AMPA-type glutamate receptor trafficking.

The effect of PtdIns(3,5)P_2_ on neurite thickness was observed in primary hippocampal neurons, cortical neurons, and CAD cells, suggesting that PtdIns(3,5)P_2_ could be a general mechanism for regulating neurite thickness. Furthermore, in addition to PIKfyve knockdown, both knockdown of NSG1 and overexpression of GFP-ML1N×2 also resulted in thinner neurites in CAD cells. The presence of the same phenotype in multiple systems after distinct molecular interventions supports the notion that neurite thickness is physiologically regulated. This neurite thinning phenotype was unlikely due to impaired neuron viability (54). Stable shPIKfyve CAD cells could be passaged and revived from frozen stocks similar to parental cells, suggesting that their viability was unaffected. Exact molecular details for the regulation of neurite thickness by PtdIns(3,5)P_2_ are still under investigation. It is likely that cytoskeleton and adhesion proteins are modulated by PtdIns(3,5)P_2_ and NSG1. Consistent with this notion, PtdIns(3,5)P_2_ was shown to be involved in the recycling of tight junction proteins claudin-1/2 in MDCK cells (76), while NSG1 is important for the trafficking of NgCAM/L1 adhesion molecules (35, 39). Further study on this topic will significantly deepen our understanding of neuron morphogenesis.

To date, how NSG1 exerts its function on endosomes still remains unclear, but it is known to be a very dynamic protein. It has been reported that NSG1 is not a resident protein on endosomes but travels rapidly along the endocytic pathway (58). We showed that treatment with PIKfyve inhibitors delayed NSG1 trafficking in EEA1-positive endosomes. This likely reduced the motility of NSG1. In a previous study, an estimate of 63% of NSG1+/EEA1-vesicles were motile, while only 8% of NSG1+/EEA1+ endosomes were motile (77). This notion is consistent with another study showing that PIKfyve is important for the motility of late endosomes/lysosomes in neurites (78). Interestingly, neurites were often ill-developed in cells with defective NSG1 localization (Fig. 4), suggesting that the transport of NSG1 is linked with neurite development.

Three types of perturbation to PtdIns(3,5)P_2_ were used in this study (in the order of phenotype severity): overexpression of Fig4 or ML1N×2, pharmacological inhibition of PIKfyve activity, and shRNA knockdown of PIKfyve expression. A serendipitous discovery was that overexpressing Fig4 or ML1N×2 had a more significant influence than PIKfyve inhibitors on mCherry-NSG1 localization. While PIKfyve inhibitors mainly caused enlarged mCherry-NSG1 vesicles, overexpressing Fig4 or ML1N×2 led to mislocalization of mCherry-NSG1 to nonendosomal locations. Accordingly, additional expression of mCherry-NSG1 rescued neurite phenotype in inhibitor-treated CAD cells (Fig. 6), but apparently not in ML1N×2/Fig4–overexpression cells (Fig. 4). The inter-conversion between phosphoinositide species is well known to coordinate membrane trafficking events both spatially and temporally (79). It is conceivable that NSG1 functions in a sequence of events that rely on the correct timing of PtdIns(3,5)P_2_ synthesis and turnover. The molecular identity of such events awaits further research. Another example supporting the importance of PtdIns(3,5)P_2_ dynamics comes from MTMR2 and Fig4, causal genes for Charcot-Marie-Tooth disease type 4B and 4J, respectively. Loss of either MTMR2 or Fig4 results in neuronal demyelination, while PtdIns(3,5)P_2_ levels were increased in the former but decreased in the latter (14). This example emphasizes that correct PtdIns(3,5)P_2_ dynamics could be as critical as its absolute levels.

It was also intriguing that overexpression of Fig4, but not Vac14, resulted in NSG1 mislocalization to nonendosomal structures. In the PtdIns(3,5)P_2_ biosynthesis complex, PIKfyve: Fig4: Vac14 is present at a 1:1:5 ratio (4). Hence, Fig4 function might be more sensitive to changes in expression than Vac14. In addition, Fig4 is involved in both the biosynthesis and turnover of PtdIns(3,5)P_2_ (4, 61, 62), which likely requires more delicate control of expression levels. Along the same line, it was reported that overexpression of Fig4 potentiated the aggregation of Lewy body–associated Synphilin-1 protein, while overexpression of Vac14 alleviated the aggregation (34).

CAD cells provide a unique “on-demand” model system for studying neurite growth. Neurite growth can be initiated after a specific molecular intervention such as PIKfyve knockdown and overexpression of ML1N×2, which is not possible in primary neurons and helps exclude the influence of cell viability. CAD cells also provide a simplified system to support *in vivo* findings. Consistent with our conclusion, it has been reported that axon calibers of optic nerves in Fig4^−/−^ mice were shifted to the smaller end (80). Note that this *in vivo* finding alone could be explained by either neurite growth defect or myelination defect in Fig4^−/−^ animals. Axon caliber and myelination regulation are intertwined, as axon caliber is increased by myelination (81), while the degree of myelination is correlated with axon caliber (80). In this case, CAD cells help delineate the cause and effect between PtdIns(3,5)P_2_ and neuron morphology. The limitation of CAD cells is that it cannot be used to study electrophysiological responses due to the lack of axon/dendrite differentiation. It remains to be shown whether enhancing the function of the NSG1 pathway could rescue PtdIns(3,5)P_2_-related defects in animals and humans.

A possible limitation of this study is that the mCherry tag and/or expression levels of mCherry-NSG1 could alter the function or dynamics of NSG1 trafficking. In this regard, the fact that mCherry-NSG1 rescued neurite thickness defects in PtdIns(3,5)P_2_-impaired neurons suggests that this protein was indeed functional. In addition, the correlation between mCherry-NSG1 mislocalization and deformed neurite morphology in Fig4 and ML1N×2 overexpression cells indicates that the transport of this tagged protein is tightly linked to neurite growth.

In summary, we identified neurite thickness as a novel physiological parameter regulated by PtdIns(3,5)P_2_. We further found that NSG1, a neuronal-specific endosomal protein, functions downstream of PtdIns(3,5)P_2_ and supports neurite morphogenesis. Our results indicate that the transport of NSG1 contributes to the sensitivity of neurons to PtdIns(3,5)P_2_ and this pathway warrants further studies as a target to alleviate PtdIns(3,5)P_2_-related neuropathies.

## Experimental procedures

### Cell culture

All animal protocols were approved by the Animal Care and Use Committee at Soochow University. Mouse hippocampal neurons were cultured as described in (30). CAD cells were a gift from Dr Kristen Verhey at the University of Michigan and cultured in DMEM/F-12 (Life Technologies, C11330500BT)–based medium supplemented with 10% fetal bovine serum (FBS, Biological Industries, 04-001-1a-us), 1% GlutaMAX (Life Technologies, 35050061), and 1% Penicillin-Streptomycin (Life Technologies, 15140122). HeLa and HEK293T cells were gifts from Dr Lois Weisman at the University of Michigan and cultured in Dulbecco’s modified Eagle’s medium (Invitrogen C11965500BT)–based medium supplemented as above. PC12 cells were obtained from the Institute of Neuroscience at Soochow University and cultured in RPMI (Life Technologies, C22400500BT)-based medium supplemented as above.

For primary cortical neurons culture, layer V sensorimotor cortex of E18.5 embryos of CD1 mice were dissociated with 0.25% trypsin for 10 min, then filtered and pelleted at 1000 rpm for 5 min, washed with 1 × PBS, and resuspended in neurobasal medium (Invitrogen, 21103049) supplemented with 5% FBS, 1% Penicillin-Streptomycin, 1% B27 (Invitrogen 17504044), and 1% GlutaMAX. Neurons were seeded in 24-well plates coated with 20 ng/ml poly-D-lysine. After 24 h, the culture medium was replaced with neurobasal medium supplemented with 2% B27, 1% Penicillin-Streptomycin, and 1% GlutaMAX.

For inhibiting PIKfyve activity, YM201636 (Selleck, S1219) and apilimod (MedChem Express, HY-14644) were applied for 12 h before fixation at 1.6 μM and 1 μM, respectively, unless indicated otherwise.

### Plasmids and antibodies

Human NSG1 gene was amplified from cDNA and cloned into XhoI/BamHI sites of pmCherry-C1 vector using the following primers: 5′-CTC GCT CGA GGA ATG GTG AAG TTG GGG AAC-3′ (forward) and 5′-GGA TGG ATC CC TAA GCT GAC TTC TCA GC-3′ (reverse). The 3xFLAG-tagged NSG1 was cloned into BamHI/EcoRI sites of pcDNA3.1 using the following primers: 5′-cgc GGA TCC atg GAC TAC AAA GAC CAT GAC GGT GAT TAT AAA GAT CAT GAT ATC GAT TAC AAG GAT GAC GAT GAC AAG GGC gtg aag ttg ggg aac aat ttc gc-3′ (forward) and 5′-ccg GAA TTC cta agc tga ctt ctc agc cgc-3′ (reverse). Citrine-Vac14 and Citrine-Fig4 were described in (30). Cit-Fig4^C486S^ plasmid was cloned from Citrine-Fig4 using site-directed mutagenesis with the following primers: 5′-GAC TGG CAT CCT TCG AAC CAA CTC TGT GGA CTG TTT AGA TCG CAC-3′ (forward) and 5′-GTG CGA TCT AAA CAG TCC ACA GAG TTG GTT CGA AGG ATG CCA GTC-3′ (reverse). GFP-Rab4, GFP-Rab6, and GFP-Rab9 were described in (82). GFP-EEA1, GFP-TRPML1, GFP-ML1N×2, GFP-ML1N^AA^×2 were gifts from Dr Haoxing Xu at the University of Michigan and described in (63). All plasmids were confirmed by Sanger sequencing.

The following antibodies were used: rabbit anti-Map2 (Millipore, AB5622), mouse anti-Tau (Sigma-Aldrich, MAB3420), mouse anti-Rab5 (Cell Signaling Technology, 46449), rabbit anti-FLAG (Beyotime, AF0036), rabbit anti-GFP/Citrine (Genescript A01704), mouse anti-EEA1 (R&D, MAB8047), mouse anti-CD63 (Biolegend, 143902), rat anti-LAMP1 (Santa Cruz, sc-19992), mouse anti-Tuj1 (Beyotime, AT809), and mouse anti-NSG1 (Santa Cruz, sc-390654).

### Transfection

CAD cells were seeded at 4 × 10^5^ cells per 35 mm dish in culturing medium without antibiotics. After 24 h, cells were transfected in the absence of FBS with 1 mg/ml PEI MAX (Polysciences Inc, 24765) at the ratio of 2:8 (μg plasmids: μl PEI MAX) diluted in Opti-MEM. After incubation for 4 to 6 h, the transfection medium was replaced with fresh media for another 24 h before being changed into the differentiation medium (full medium without FBS). CAD cells were differentiated for 36 to 48 h, followed by fixation for microscopy.

Primary cortical neurons were transfected with lipofectamine 2000 (Invitrogen, 11668027) according to the manufacturer's instruction at 2 div and used for downstream experiments at 24 h after transfection.

### Microscopy

For fluorescent protein (Citrine, GFP, mCherry)–based imaging, cells expressing fluorescent proteins were fixed by 3.7% paraformaldehyde for 15 min, washed with PBS, and incubated with 0.1 μg/ml DAPI (Beyotime, C1005) for 5 min. Slides were mounted with 10 mg/ml propyl gallate in 50% PBS/50% glycerol and imaged with a Leica TCS SP8 confocal microscope using a 60× oil-immersed objective (N.A. = 1.4).

For live cell imaging, CAD cells were grown on glass-bottom culture dishes and transfected with fluorescent proteins for 24 h, followed by differentiation for another 24 h. Cells were changed into Hepes-buffered phenol-red free medium, placed in a heated humidity chamber (Tokai Hit), and imaged as above. For live imaging experiments with PIKfyve inhibitors, the imaging was started at 30 min after inhibitor addition to minimize the effect of phototoxicity.

For immunofluorescence, fixed cells were permeabilized with 0.1% Triton X-100 for 5 min, washed with PBS, and then incubated with blocking solution (2 % normal goat serum in PBS) for 1 h. After blocking, cells were incubated with primary antibody diluted in the blocking solution (anti-Rab5, 1:200; 1:100 for all other antibodies in this study) for overnight at 4 °C, washed with PBS, followed by incubation with secondary antibodies (Alexa 488 goat anti-mouse for Rab5 and Tuj1; Alexa 488 goat anti-rabbit for Map2; Alexa 555 goat anti-mouse for Tau and NSG1, Alexa 647 goat anti-mouse for EEA1 and CD63; Alexa 647 goat anti-rat for LAMP1, 1:200 in blocking solution). Slides were mounted and imaged as above.

### RNA interference

Lentiviral plasmids (pLKO.1-puro based, encoding puromycin resistance and no fluorescent protein) containing shRNA against mouse PIKfyve gene and nontargeting control shRNA were described in (29). Lentivirus particles were packaged in HEK293T cells by cotransfection with pMD2G and psPAX2 plasmids at the following ratio: 1 μg viral vector, 0.5 μg pMD2G, and 0.75 μg psPAX2. Supernatant media were collected at 36 to 72 h and filtered with 0.45 μm filters (Merck, SLHV033RB). For infection, CAD cells were incubated with virus-containing media mixed with fresh medium at 1:1 ratio, together with 4 μg/ml polybrene for 24 h. After growth for another 24 h, infected cells were selected with 2 μg/ml puromycin for 2 to 3 days until> 90% of control cells were killed. Stably infected cells were maintained in 2 μg/ml puromycin-containing medium during growth and differentiation and used within 1 month.

Plasmids encoding shRNA against mouse NSG1 were designed and cloned by GenePharma Inc. The plasmid backbone used was pGPU6/GFP/Neo, which also encoded GFP to help identify transfected cells. The following targeting sequences were used: shNSG1-800: GCT TCG ACA CCA TTC CTT TGA; shNSG1-1073: GCC CTG ATG GGT TTG TCT TGA; shNSG1-1125: GAG CTA CTA CAC GGA GCA AGA; nontargeting control: GTT CTC CGA ACG TGT CAC GT. CAD cells were transiently transfected with shRNA-expressing plasmids, differentiated for 24 h, and fixed for imaging.

Knockdown efficiency was analyzed by quantitative real-time PCR. RNA was extracted with Total RNA Extractor (Sangon, B511311) according to the manual and reverse transcribed with PrimeScript RT Master Mix (Takara, RR036A). Real-time PCR was performed using SYBR Premix EX Taq II enzyme mix (Takara, RR820A) on an ABI 7500 Real-Time PCR system (Applied Biosystems). Mouse PIKfyve mRNA expression was detected with primer pairs: 5′-CCG GCG CTC TTC AGT GTT AG-3′ (forward) and 5′-GAG GCG TTT CAA TAC TGT GCT-3′ (reverse). Mouse NSG1 was detected with primer pairs: 5′- GGC TTC GAC ACC ATT CCT TTG-3′ (forward) and 5′- TTT TCA CCA CGA CCT TAT CTG G-3′ (reverse). Mouse L32 expression was used as internal reference: 5′-TTA AGC GAA ACT GGC GGA AAC-3′ (forward) and 5′-TTG TTG CTC CCA TAA CCG ATG-3′ (reverse). Relative gene expression was calculated with the 2^−ΔΔCt^ method.

### Coimmunoprecipitation

HEK293T cells were transfected with 3xFLAG-NSG1 and Cit-Vac14 or Citrine control plasmids. After 24 h, cells were scraped and homogenized by forcing through a 22-gauge needle several times in lysis buffer (10 mM Hepes, 100 mM NaCl, 1 mM EDTA, 1× Proteinase inhibitor cocktail). Crude extracts were spun down at 14, 000 rpm for 20 min at 4 °C. The supernatant was incubated with washed Anti-FLAG M2 affinity gel (Sigma-Aldrich, A2220) overnight at 4 °C. The resin was washed three times by spinning down at 2000 g for 3 min and incubation with 1:1 mixed lysis buffer and PBS. Immunoprecipitated proteins were eluted by heating in Laemmli sample buffer at 95 °C for 5 min and spun down at 2000 g. Proteins were separated by SDS-PAGE electrophoresis and transferred to PVDF membrane. The membrane was blocked with 5% nonfat milk and incubated with primary antibodies (rabbit anti-FLAG or rabbit anti-GFP, 1: 1000 in the blocking buffer) overnight at 4 °C, followed by washing and incubation with HRP-conjugated goat anti-rabbit secondary antibodies (1:1000). The membrane was developed by Immobilon Western Chemiluminescent HRP Substrate (Millipore, WBKLS0500).

### Neurite thickness and colocalization analysis

Primary hippocampal neurons were immunostained with anti-Map2 and anti-Tau to label dendrites and axons, respectively. CAD neurons were transfected with Citrine as a volume marker to delineate neurites. Primary cortical neurons were labeled with anti-Tuj1 to identify neurites. At least five random fields were imaged for each coverslip. Neurite diameter was measured on individual neurons pooled from two to three independent experiments. As neurites taper gradually from the base toward the tip, the diameter was measured at the proximal segment of the thickest neurite with ImageJ’s line tool manually. Neurite diameters in different conditions were compared using nonparametric tests, which do not require the assumption of normality. For two-group comparisons, Mann-Whitney U test was performed. For three-group comparisons, Kruskal-Wallis test was performed, followed by post hoc Dunn’s multiple comparisons test.

Colocalization was measured on images of single z-planes using the JACoP plugin of ImageJ. Images were autothresholded. Manders’ coefficient was used, which indicates the fraction of red pixels that overlaps with the green channel or the fraction of green pixels that overlaps with the red channel. Normality of the colocalization coefficient was not assumed, and nonparametric tests were used as above for comparison among different groups.

### Statistics

Statistical analysis and graphing were carried out with Prism 9 (GraphPad). Statistical tests used were indicated in the figure legends. Student’s *t* test and one-way ANOVA were used for parametric testing. Kruskal-Wallis test and Mann-Whitney U test were used for nonparametric testing. Significance levels were set at *p* < 0.05. Data were shown as mean ± (SD).

## Data availability

All data are contained within this article and in the supporting information.

## Supporting information

This article contains supporting information.

## Conflict of interest

The authors declare that they have no conflicts of interest with the contents of this article.

## Supporting information

Videos S1–S7
