# Supplementary material for: Phosphatidylinositol (3,5)-bisphosphate machinery regulates neurite thickness through neuron-specific endosomal protein NSG1/NEEP21
Source: J Biol Chem. 2022 Dec 7;299(1):102775. doi: 10.1016/j.jbc.2022.102775 (PMC9823133; doi:10.1016/j.jbc.2022.102775)
Supplement: Supplemental Figures S1–S7 [file mmc8.pdf]

Supporting information

Phosphatidylinositol (3,5)-bisphosphate machinery regulates neurite thickness through neuron-specific endosomal protein NSG1/NEEP21

Lijuan Qi[1,2], Chen Sun[3], Shenqing Sun[1], Aiqing Li[1], Qiuming Hu[1], Yaobo Liu[3], Yanling Zhang[1*]

[1]Department of Biochemistry and Molecular Biology, Soochow University Medical College, Suzhou, Jiangsu 215123, China

[2]National Clinical Research Center for Hematologic Diseases, Jiangsu Institute of Hematology, the First Affiliated Hospital of Soochow University, Suzhou, Jiangsu 215123, China

[3]Jiangsu Key Laboratory of Neuropsychiatric Diseases and Institute of Neuroscience, Soochow University; Clinical Research Center of Neurological Disease, The Second Affiliated Hospital of Soochow University, Suzhou, Jiangsu 215123, China

* To whom correspondence should be addressed: Yanling Zhang; Department of Biochemistry and Molecular Biology, Soochow University Medical College, Suzhou, Jiangsu, 215123, China; yanlzhan@suda.edu.cn Tel:86-512-65880108

Running title: PtdIns(3,5)P_2 regulates neurite thickness

List of supporting information:
Figures S1-S7
Videos S1-S7

Supplemental Video Legends

Video S1. mCherry-NSG1 dynamics in CAD cells. CAD cells were transfected with mCherry-NSG1 and differentiated for 24 hours. Time series were acquired at one frame per 10 seconds. Playback was set at 7 frames per second.

Video S2. mCherry-NSG1 colocalizes with GFP-TRPML1 in CAD cells. CAD cells were transfected with mCherry-NSG1 (red) and GFP-TRPML1 (green), and differentiated for 24 hours. Time series were acquired at one frame per 30 seconds. Playback was set at 7 frames per second.

Video S3-S4. mCherry-NSG1 vesicles are enlarged after PIKfyve inhibitor treatment. CAD cells were transfected with mCherry-NSG1, differentiated for 24 hours, and treated with 1.6 μM YM201636 (Video S3), or 1 μM apilimod (Video S4). Time series were acquired at one frame per minute. Playback was set at 4 frames per second.

Video S5-S7. mCherry-NSG1 vesicles are accumulated in EEA1-positive endosomes after PIKfyve inhibitor treatment. CAD cells were transfected with mCherry-NSG1 (red) and GFP-EEA1 (green), differentiated for 24 hours, and treated with DMSO control (Video S5), 1.6 μM YM201636 (Video S6), or 1 μM apilimod (Video S7). Time series were acquired at one frame per 30 seconds. Playback was set at 7 frames per second.

Figure S1

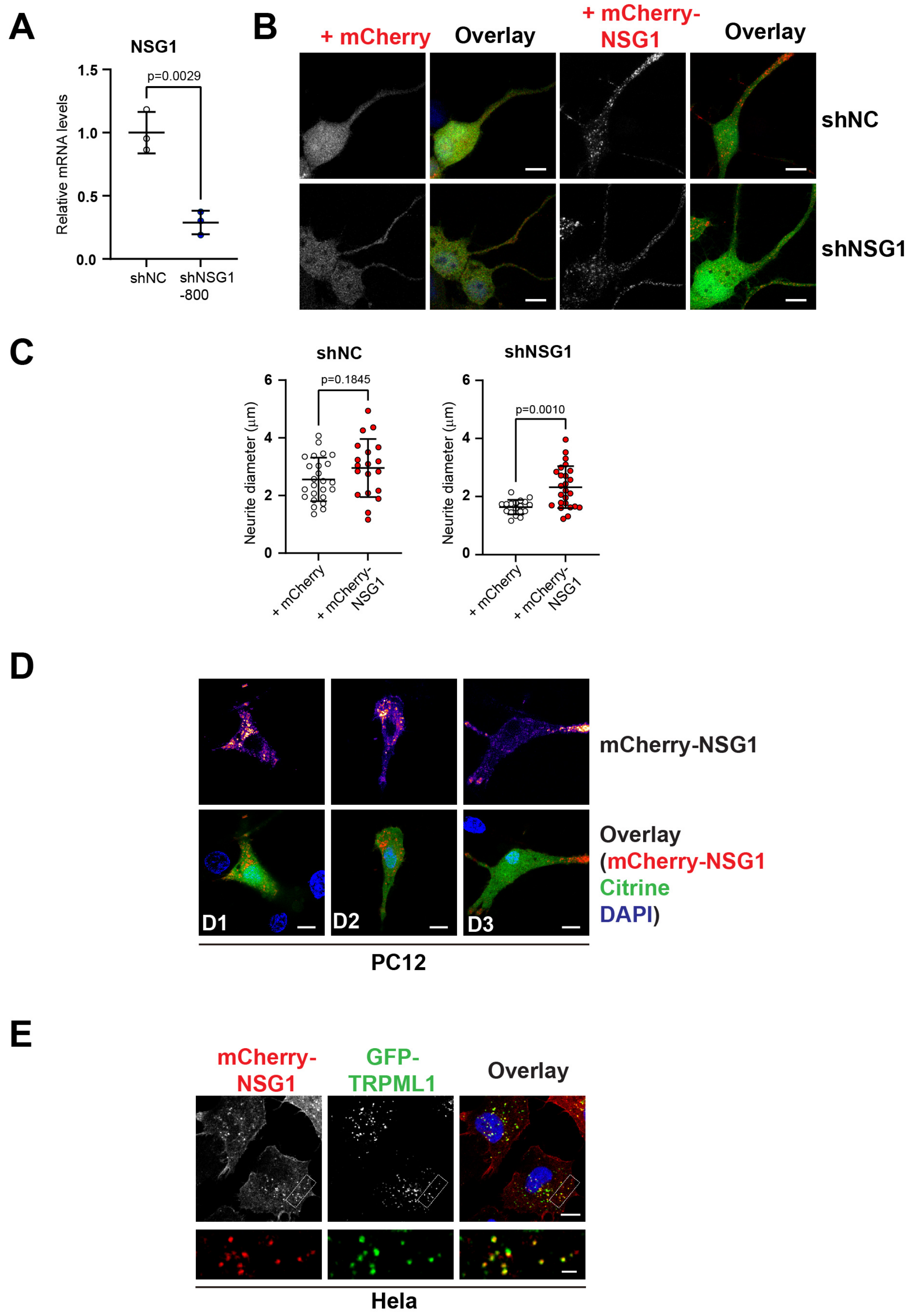

Figure S1. NSG1 is involved in neurite thickness and localizes to PI(3,5)P_2-enriched vesicles.

A. CAD cells were infected with lentiviruses expressing non-targeting control shRNA (shNC) or shRNA against mouse NSG1 (shNSG1-800 in Figure 3). The expression of NSG1 mRNA was determined by qRT-PCR. N = 3 independent experiments. Significance was determined by Student's t-test.

B. CAD cells with shNC or shNSG1 expression were transfected with mCherry or mCherry tagged human NSG1, which is resistant to the shNSG1-800 targeting. GFP is encoded on the lentivirus vector backbone. Scale bars, 10 μm.

C. Quantitation of neurite thickness in **B.** N = 18-25 cells from two independent experiments. Significance was determined by Mann-Whitney test.

D. Distribution of mCherry-NSG1 in the soma and neurites in PC12 cells. PC12 cells were transfected with mCherry-NSG1 and Citrine. Panels D1, D2, and D3 represent various localization patterns of NSG1. Scale bars, 10 μm.

E. Hela cells were transfected with mCherry-NSG1 (red) and GFP-TRPML1 (green). Yellow puncta represent the colocalization of the two proteins. Scale bars, 10 μm for the upper panel and 2 μm for the zoomed insets.

Error bars, mean ± SD.

Figure S2

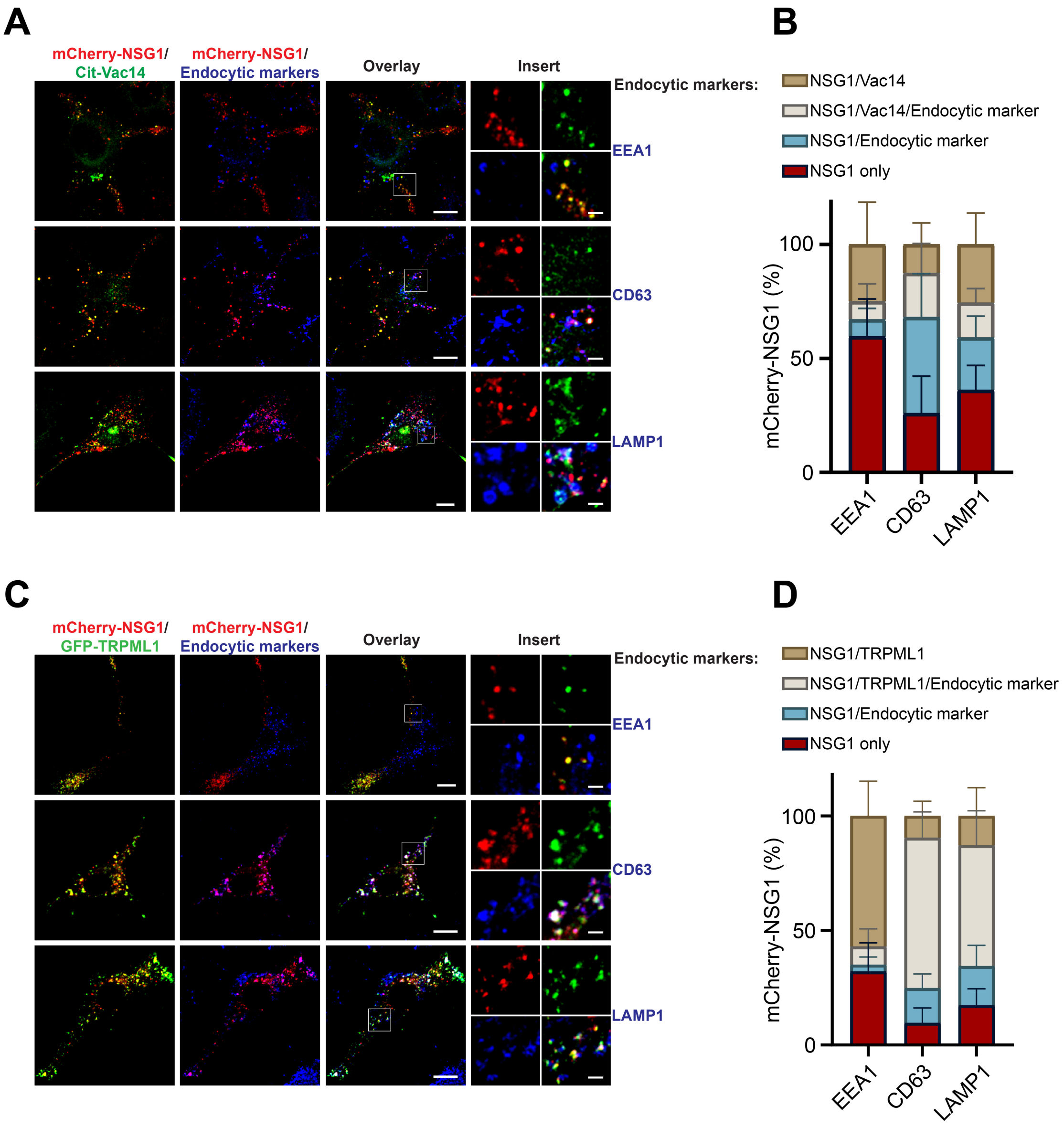

Figure S2. mCherry-NSG1 colocalizes with Cit-Vac14 and GFP-TRPML1 along the endocytic pathway.

A-D. CAD cells were transfected with mCherry-NSG1 and Cit-Vac14 (**A-B**) or GFP-TRPML1 (**C-D**), followed by fixation and immuno-labeling with endocytic markers, including anti-EEA1, anti-CD63 or anti-LAMP1. To quantify colocalization, mCherry-NSG1 puncta were classified into four categories: mCherry-NSG1 alone, mCherry-NSG1 that colocalized with Cit-Vac14 (**B**) or GFP-TRPML1 (**D**) only, mCherry-NSG1 that colocalized with the endocytic marker of interest only, mCherry-NSG1 that colocalized with both Cit-Vac14 (**B**) or GFP-TRPML1 (**D**) and the endocytic marker of interest. N = 10-15 cells for each category. Scale bars in (**A**) and (**C**), 10 µm for the main panels and 2 µm for the zoomed insets. Error bars, SD.

Figure S3

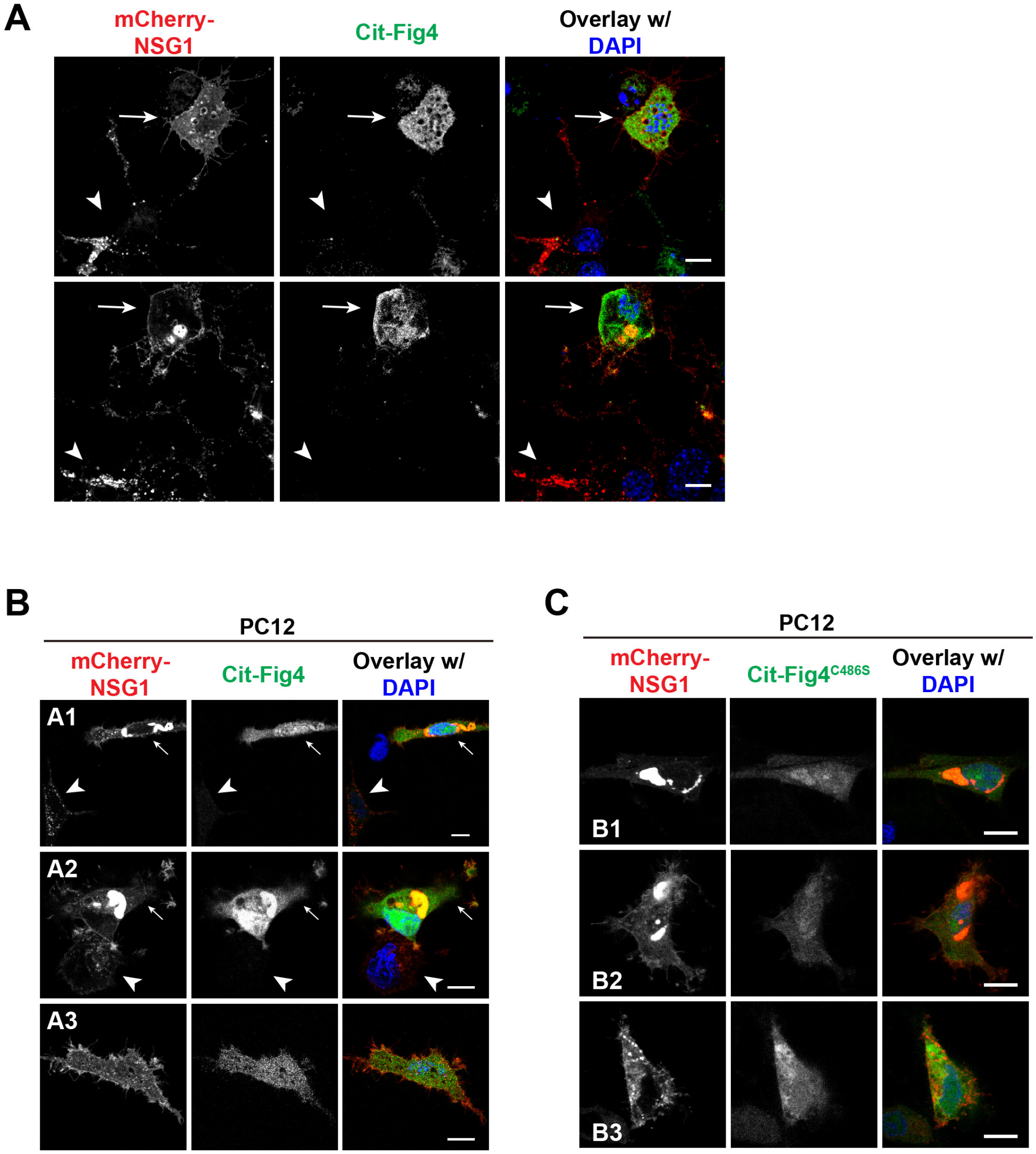

Figure S3. NSG1 is mis-localized by Cit-Fig4 overexpression.

A. CAD cells were transfected with mCherry-NSG1 and Cit-Fig4. Cells expressing only mCherry-NSG1 but no Cit-Fig4 expression (arrowheads) had normal punctate mCherry-NSG1 localization. Cells expressing both mCherry-NSG1 and Cit-Fig4 (arrows) had mis-localized mCherry-NSG1. The latter cells are also shown in Figure 4.

B-C. PC12 cells were transfected with mCherry-NSG1 and Cit-Fig4 (**B**) or Cit-Fig4^{C486S} (**C**). Cells expressing only mCherry-NSG1 but no Cit-Fig4 expression (arrowheads) had normal punctate mCherry-NSG1 localization, while cells expressing both mCherry-NSG1 and Cit-Fig4 (arrows) had

mis-localized mCherry-NSG1 as in Figure 4.
Scale bars, 10 μm.

Figure S4

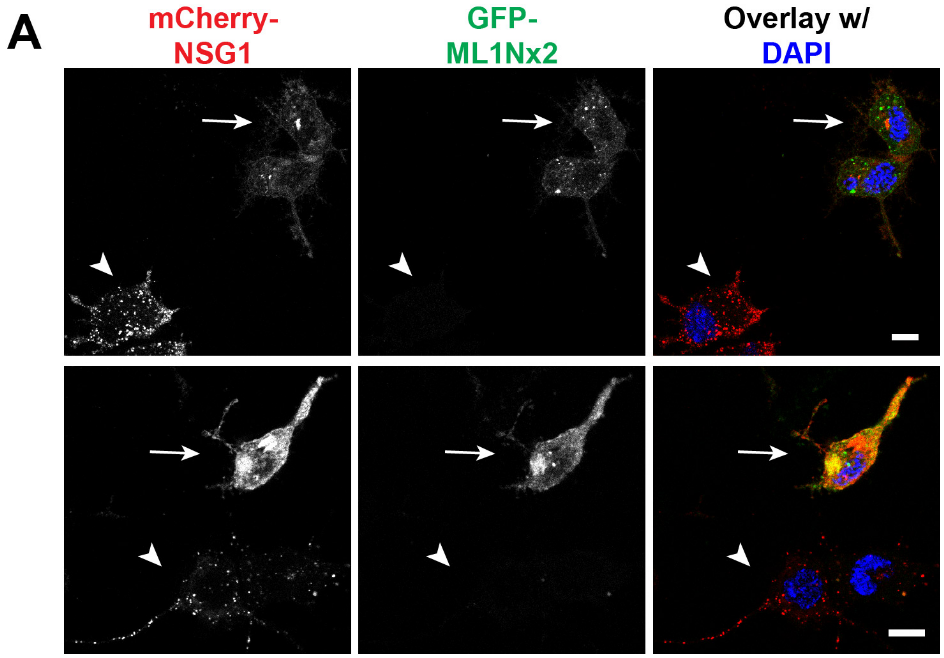

Figure S4. NSG1 is mis-localized by GFP-ML1Nx2 and Cit-Fig4.

A. CAD cells were transfected with mCherry-NSG1 and GFP-ML1Nx2. Cells expressing only mCherry-NSG1 and no GFP-ML1Nx2 (arrowheads) had normal punctate mCherry-NSG1 localization, while cells expressing both mCherry-NSG1 and Cit-Fig4 (arrows) had mis-localized mCherry-NSG1. The latter cells are also shown in Figure 4.

Scale bars, 10 μm.

Figure S5

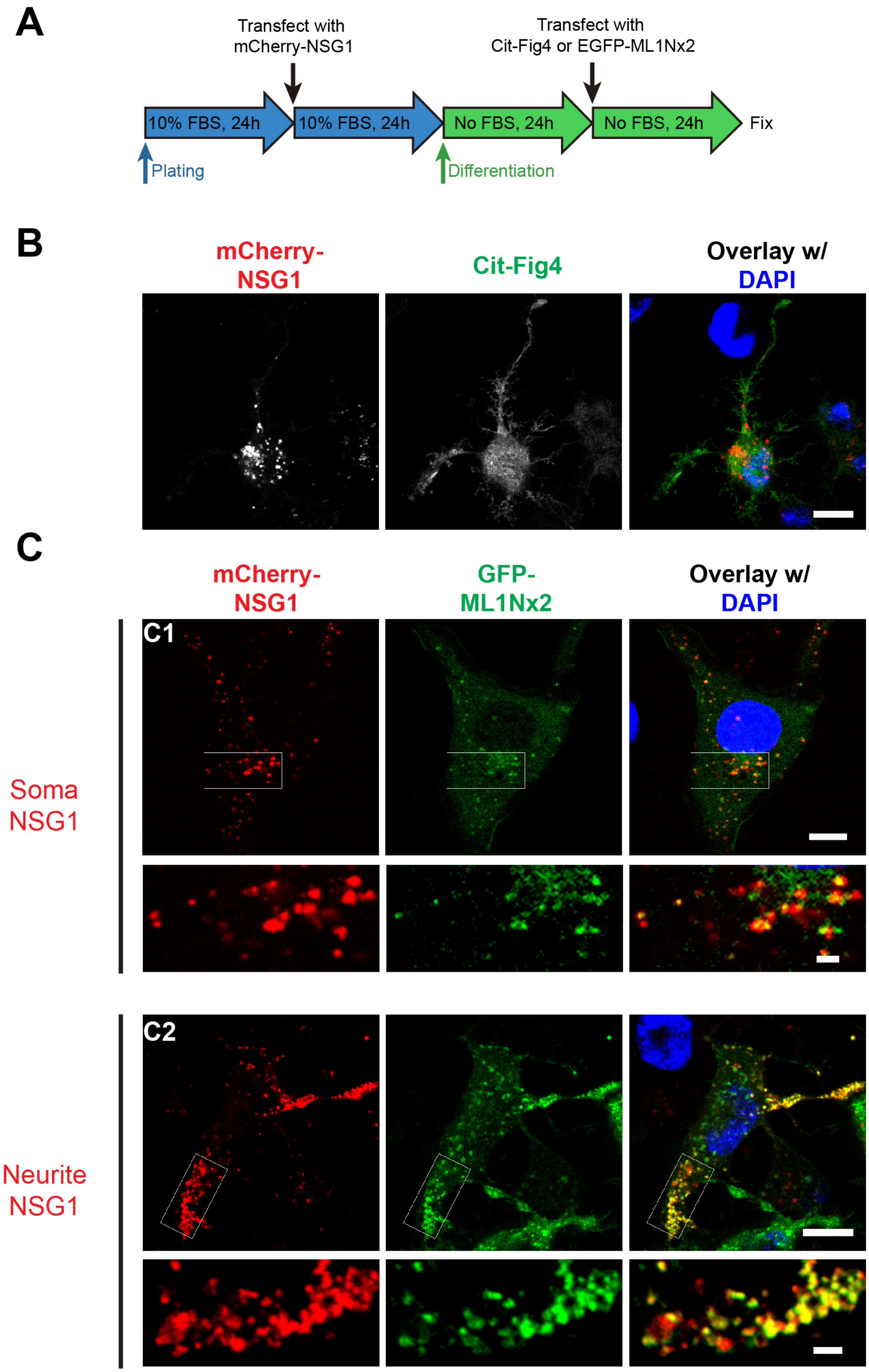
A
Transfect with mCherry-NSG1
Transfect with Cit-Fig4 or EGFP-ML1Nx2
10% FBS, 24h
10% FBS, 24h
No FBS, 24h
No FBS, 24h
Fix
Plating
Differentiation
B
mCherry-NSG1
Cit-Fig4
Overlay w/ DAPI
C
mCherry-NSG1
GFP-ML1Nx2
Overlay w/ DAPI
C1
Soma NSG1
C2
Neurite NSG1

Figure S5. A brief expression of Cit-Fig4 or GFP-ML1Nx2 does not mis-localize NSG1.

A. Transfection scheme. CAD cells were transfected with mCherry-NSG1. After 24 hours of expression, cells were differentiated for another 24 hours and transfected with Cit-Fig4 (**B**) or GFP-ML1Nx2 (**C**).

B. mCherry-NSG1 proteins still had punctate localization when Cit-Fig4 was briefly expressed. Scale bars, 10 μm.

C. mCherry-NSG1 co-localized with briefly expressed GFP-ML1Nx2 in both soma (panel C1) and neurites (panel C2). Scale bars, 10 μm for the main panels and 2 μm for the zoomed insets.

Figure S6

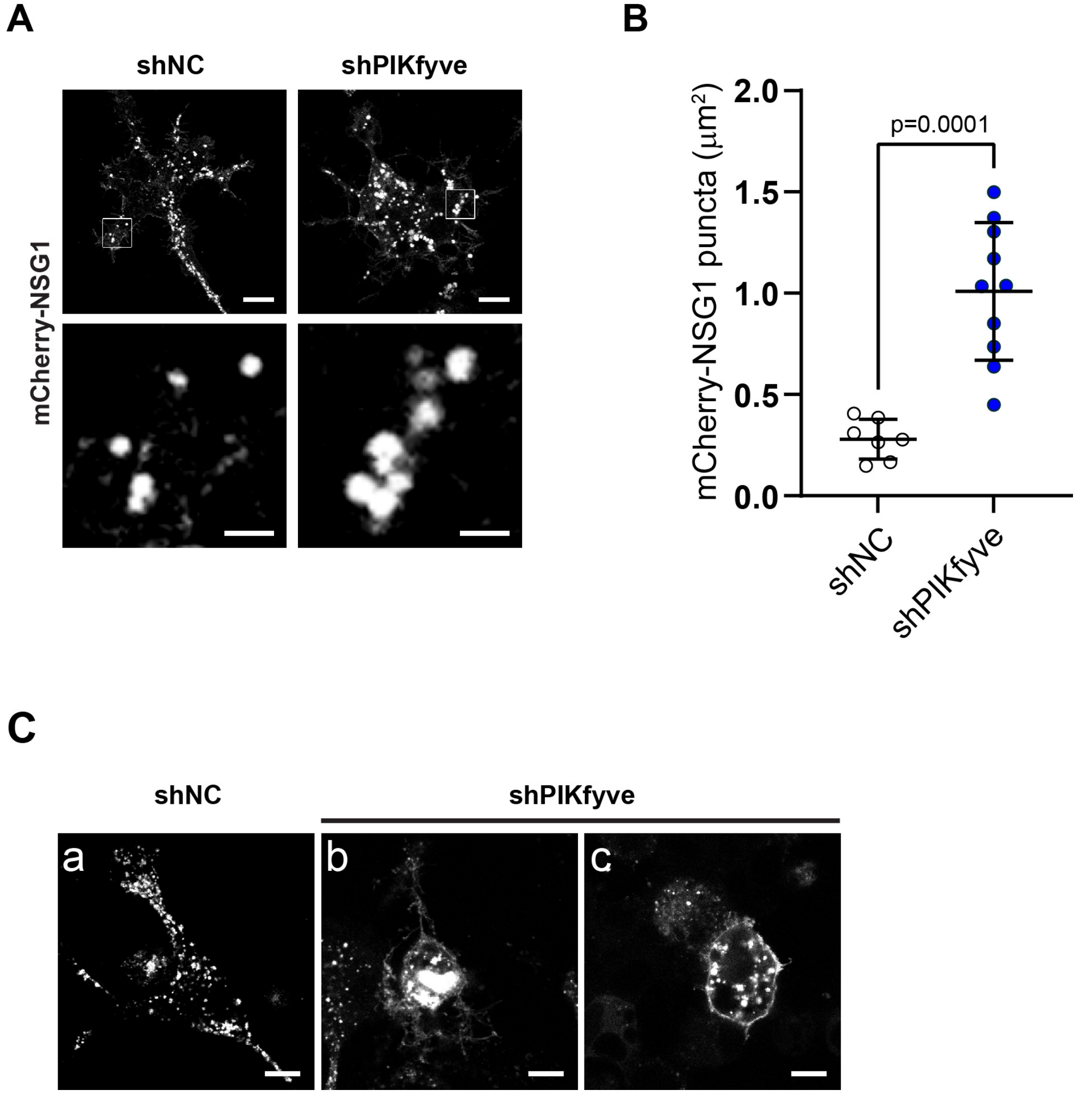

Figure S6. mCherry-NSG1 localization in stable PIKfyve knockdown CAD cells.

A. CAD cells with stable shNC or shPIKfyve expression were transfected with mCherry-NSG1. Scale bars, 10 μm for the upper panel and 2 μm for the zoomed insets.

B. Quantitation of the size of mCherry-NSG1 vesicles in shNC and shPIKfyve cells. N = 7-10 cells. with ~ 40-200 vesicles per cell. The average vesicle size of each cell was calculated. Significance was determined by Mann-Whitney test. Error bar, mean ± SD.

C. Selected cells with perinuclear (panel b) or surface (panel c) mCherry-NSG1 in shPIKfyve cells were shown. Scale bars, 10 μm.

Figure S7

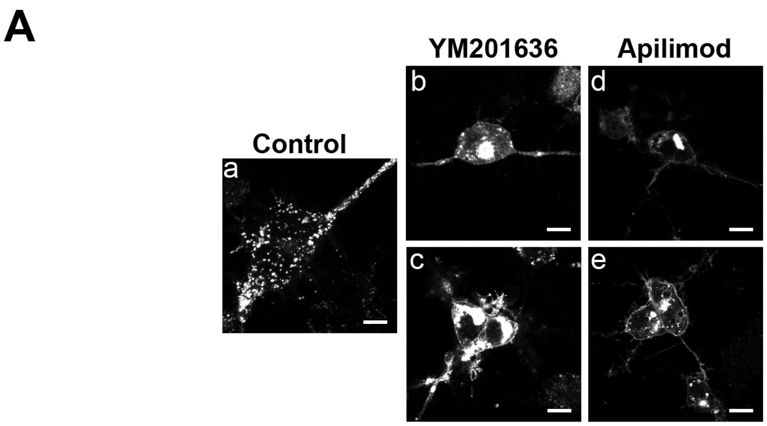
A
YM201636
Apilimod
Control
a
b
d
c
e

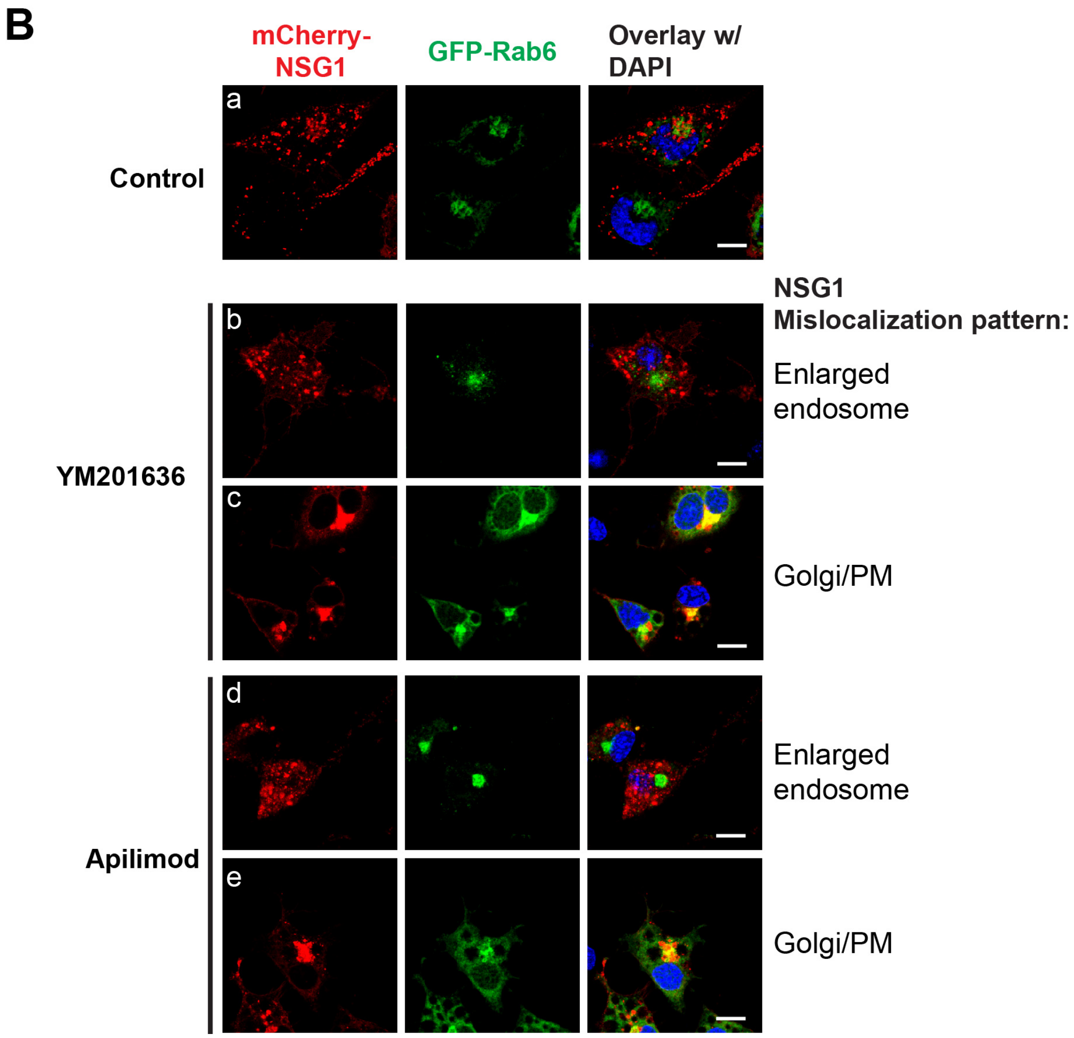
B
mCherry-NSG1
GFP-Rab6
Overlay w/ DAPI
Control
a
NSG1 Mislocalization pattern:
YM201636
b
Enlarged endosome
c
Golgi/PM
Apilimod
d
Enlarged endosome
e
Golgi/PM

Figure S7. NSG1 protein is mis-localized in selected cells after suppressing PIKfyve activity.

A. CAD cells expressing mCherry-NSG1 were treated with DMSO (Control), 1.6 μM YM201636, or 1 μM apilimod for 12 hours. Selected inhibitor-treated cells with perinuclear (panel b and d) or surface mCherry-NSG1 (panel c and e) were shown.

B. CAD cells co-transfected with mCherry-NSG1 and GFP-Rab6 (Golgi marker) were treated by DMSO (Control), 1.6 μM YM201636, or 1 μM apilimod for 12 hours. In cells with perinuclear concentrated mCherry-NSG1, colocalization between mCherry-NSG1 and GFP-Rab6 was observed. Normal punctate mCherry-NSG1, whether enlarged or not, had little colocalization with GFP-Rab6. Scale bars, 10 μm.
